# Wnt5a promotes cancer cell invasion and proliferation by receptor-mediated endocytosis-dependent and -independent mechanisms, respectively

**DOI:** 10.1038/srep08042

**Published:** 2015-01-27

**Authors:** Kensaku Shojima, Akira Sato, Hideaki Hanaki, Ikuko Tsujimoto, Masahiro Nakamura, Kazunari Hattori, Yuji Sato, Keiji Dohi, Michinari Hirata, Hideki Yamamoto, Akira Kikuchi

**Affiliations:** 1 Department of Molecular Biology and Biochemistry, Graduate School of Medicine, Osaka University, 2-2 Yamadaoka, Suita 565-0871, Japan; 2 Diagnostics Division, Discovery Research Laboratory for Innovative Frontier Medicines, Shionogi & Co., Ltd. 1-1, Futaba-cho 3-chome, Toyonaka, 561-0825, Japan; 3 Department of Informatics & Structure-based Drug Discovery, Discovery Research Laboratory for Innovative Frontier Medicines, Shionogi & Co., Ltd. 1-1, Futaba-cho 3-chome, Toyonaka, 561-0825, Japan; 4 Department of Oncology & Immunology, Discovery Research Laboratory for Innovative Frontier Medicines, Shionogi & Co., Ltd. 1-1, Futaba-cho 3-chome, Toyonaka, 561-0825, Japan

## Abstract

Wnt5a activates the Wnt/β-catenin-independent pathway and its overexpression is associated with tumor aggressiveness enhancing invasive activity. For this action, Wnt5a-induced receptor endocytosis with clathrin is required. Wnt5a expression was previously believed to be associated with cancer cell motility but not proliferation. Recently, it was reported that Wnt5a is also implicated in cancer cell proliferation, but the mechanism was not clear. In this study, we generated a neutralizing anti-Wnt5a monoclonal antibody (mAb5A16) to investigate the mechanism by which Wnt5a regulates cancer cell proliferation. Wnt5a stimulated both invasion and proliferation of certain types of cancer cells, including HeLaS3 cervical cancer cells and A549 lung cancer cells although Wnt5a promoted invasion but not proliferation in other cancer cells such as KKLS gastric cancer cells. mAb5A16 did not affect the binding of Wnt5a to its receptor, but it suppressed Wnt5a-induced receptor-mediated endocytosis. mAb5A16 inhibited invasion but not proliferation of HeLaS3 and A549 cells. Wnt5a activated Src family kinases (SFKs) and Wnt5a-dependent cancer cell proliferation was dependent on SFKs, yet blockade of receptor-mediated endocytosis did not affect cancer cell proliferation and SFK activity. These results suggest that Wnt5a promotes invasion and proliferation of certain types of cancer cells through receptor-mediated endocytosis-dependent and -independent mechanisms, respectively.

Wnt signaling is important for various developmental processes while post-neonatal abnormalities in the signaling can result in several diseases, including cancer and bone degeneration^1^,^2^. As an extracellular ligand, Wnt activates different intracellular signaling cascades: the β-catenin-dependent and β-catenin-independent pathways^3^,^4^. In the former pathway, β-catenin is targeted for degradation after phosphorylation by the Axin complex which is composed of Axin, *adenomatous polyposis coli* gene product (APC), and glycogen synthase kinase 3 (GSK3)^5^; binding of Wnt to its receptor Frizzled (Fz) and low density lipoprotein related protein 5/6 (LRP5/6) suppresses the Axin function through caveolin-mediated receptor endocytosis, leading to the stabilization of β-catenin^3^,^6^. Cytoplasmic β-catenin translocates to the nucleus where it binds and activates the transcription factor Tcf/Lef^1^,^2^. Genetic alterations in the *APC, CTNNB1* (β-catenin), and *AXIN1* (Axin) genes are frequently observed in various types of cancer, including colorectal cancer and hepatocellular carcinoma^7^. However, the relationship between abnormalities in Wnt ligands that activate the β-catenin-dependent pathway and tumorigenicity has not yet been clarified. In contrast, Wnt5a, which activates the β-catenin-independent pathway, has been shown to exhibit elevated expression in cancer cells; elevated expression of Wnt5a is associated with progression of melanoma and lung, gastric, breast and prostate cancers^8^,^9^,^10^,^11^,^12^,^13^,^14^.

The β-catenin-independent pathway regulates cytoskeleton-mediated processes and polarity establishment by activating small G proteins, such as Rac and Rho, as well as certain protein kinases, including protein kinase C (PKC) and Ca^2+^/calmodulin kinase (CaMK)^4^,^15^. Wnt5a binds to its receptors, Fz and receptor tyrosine kinase-like orphan receptor 1/2 (Ror1/2), and induces the internalization of receptors in a clathrin-mediated manner, thereby activating Rac^15^,^16^. In melanoma, Wnt5a potentiates metastasis through the induction of epithelial mesenchymal transition in a PKC-dependent manner and expression of Wnt5a is also correlated with poor prognosis^8^,^17^. Wnt5a is expressed in tumor-associated macrophages in breast cancer^13^ and both Wnt5a and Wnt5b are highly expressed in cerebral metastases of breast cancer patients^18^. Wnt5a activates Rac and induces laminin-γ expression, thereby promoting migration and invasion in gastric cancer cells; the 5-year survival is reduced in the Wnt5a-positive gastric cancer patients^10^,^11^. Wnt5a also exhibits enhanced expression in roughly 30% of prostate cancer cases that have a high rate of relapse^14^. However, knockdown of Wnt5a did not affect gastric or prostate cancer cell proliferation either *in vitro* or *in vivo*^10^,^11^,^14^. Thus, it was previously thought that Wnt5a expression contributes to the tumor aggressiveness by enhancing cancer cell invasion and metastasis rather than by promoting cell proliferation in these cancers. Yet, it has been recently reported that Wnt5a expression is involved in proliferation of several tumor cell types, including glioma, lung cancer, and T-cell leukemia^19^,^20^,^21^, suggesting that Wnt5a promotes cancer cell proliferation in a cell-context specific manner. However, whether the mechanism that underlies Wnt5a regulation of cancer cell proliferation is similar to the mechanism by which it regulates cancer cell migration and invasion is unknown.

An anti-Wnt5a polyclonal neutralizing antibody was generated previously, which inhibits tumor metastasis, but not proliferation, of gastric cancer cells *in vivo*^22^. Because the neutralizing activity of polyclonal antibodies varies among antibody lots, the generation of a neutralizing monoclonal antibody against Wnt5a was necessary to control quality and quantity of the antibody. The generation of a mouse anti-human Wnt5a monoclonal antibody using conventional methods did not succeed, probably due to the 100% identity between the human and mouse Wnt5a amino acid sequence^23^. Therefore, an anti-Wnt5a monoclonal antibody was generated using a phage library^24^,^25^,^26^. Using the monoclonal antibody, we demonstrated that Wnt5a stimulates both invasion and proliferation in certain types of cancer cells and that these cellular functions are distinctly regulated by receptor-mediated endocytosis-dependent and -independent manners.

## Results

### Generation of an anti-Wnt5a monoclonal antibody by phage library

**Figure 1:**
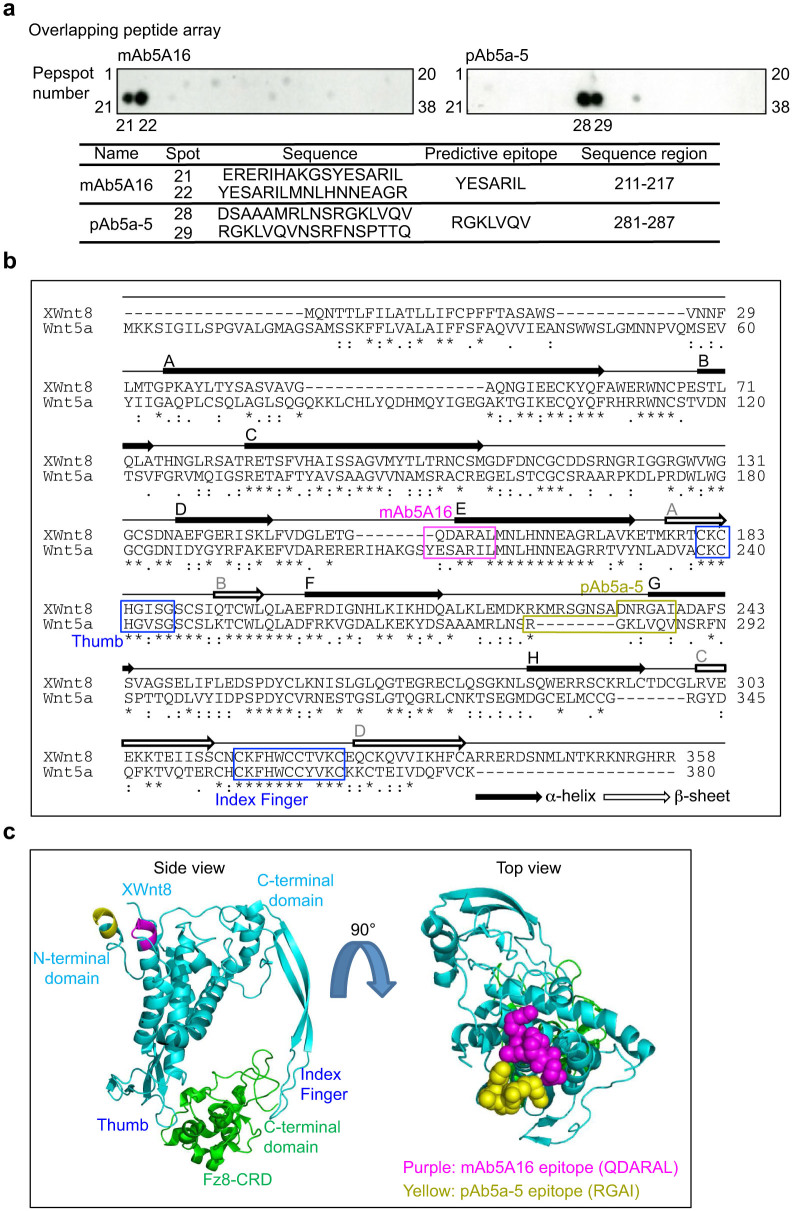
Generation of an anti-Wnt5a monoclonal antibody. (a) Pepspot membranes, in which 38 different 17-amino acid peptides were spotted, were probed with mAb5A16 and pAb5a-5. Bound antibodies were detected using a HRP-conjugated antibody. The peptide sequences recognized by the antibodies are described in the bottom panel. (b) Two distinct sites (thumb loop and index finger) of XWnt8, which interacts with Fz8, are shown on the sequence alignment of human Wnt5a. Predicted amino acid sequences for mAb5A16 and pAb5a-5 epitopes are indicated with boxes (mAb5A16, purple box; pAb5a-5, yellow box). (c) Predicted epitopes for mAb5A16 and pAb5a-5 are indicated in purple (QDARAL) and yellow (RGAI), respectively, based on the crystal structure of the XWnt8 (blue)-Fz8 (green) complex. Because three-dimensional structure of 222–234 amino acids (DKRKMRSGNSADN) in XWnt8 was not resolved, the sequence RGAI is shown instead of DNRGAI. All structural figures were prepared using the PyMOL Molecular Graphics System (Version 1.3r1).

From a 20,000 clone library, eight Wnt5a-specific clones were isolated using sequence and a phage enzyme-linked immunosorbent assay (ELISA). Treatment with the Fab fragment from *E. coli* expressing clone #16 (Fab16) resulted in the highest inhibition of KKLS gastric cancer cell invasion, which was dependent on endogenous Wnt5a (Supplementary Figure S1A); in addition, Wnt5a expression induced MKN-45 gastric cancer cell invasion and the Fab16 inhibited Wnt5a-dependent invasion (Supplementary Figure S1B). Fab16 was converted into rat IgG1 and this anti-Wnt5a monoclonal antibody was referred to as mAb5A16. mAb5A16 was expressed in and purified from HEK293 cells. The affinity of mAb5A16 for Wnt5a was almost identical to pAb5a-5, the rabbit polyclonal anti-Wnt5a antibody that we generated previously^22^ (Supplementary Figure S1C). To define the epitopes of Wnt5a recognized by mAb5A16 and pAb5a-5, 38 different 17-amino acid peptides (Pepspot), in which 7-amino acids are overlapping adjacent peptides, were generated based upon the entire human Wnt5a amino acid sequence. An epitope mapping assay showed that the predicted epitopes recognized by mAb5A16 and pAb5a-5 are the amino acids YESARIL (211–217) and RGKLVQV (281–287), respectively (Figures 1a and b).

Crystal structure analysis revealed that *Xenopus* Wnt8 (XWnt8) interacts with the extracellular domain of Fz8 through two sites^27^. One interaction (the “thumb”) is mediated by the lipid modification of XWnt8 and a groove of Fz8, and the other interaction (the “index finger”) is composed of the hydrophobic interaction between the C-terminal region of XWnt8 and a depression on Fz8 (Figure 1c). Given that human Wnt5a shows 67% amino acid similarity to XWnt8, the predicted peptides recognized by mAb5A16 and pAb5a-5 were superimposed on the three dimensional structure of XWnt8 (Figure 1c). The spatial positions of epitopes of mAb5A16 and pAb5a-5 were located close together (Figure 1c).

### mAb5A16 blocks receptor-mediated endocytosis and inhibits cell migration and invasion

**Figure 2:**
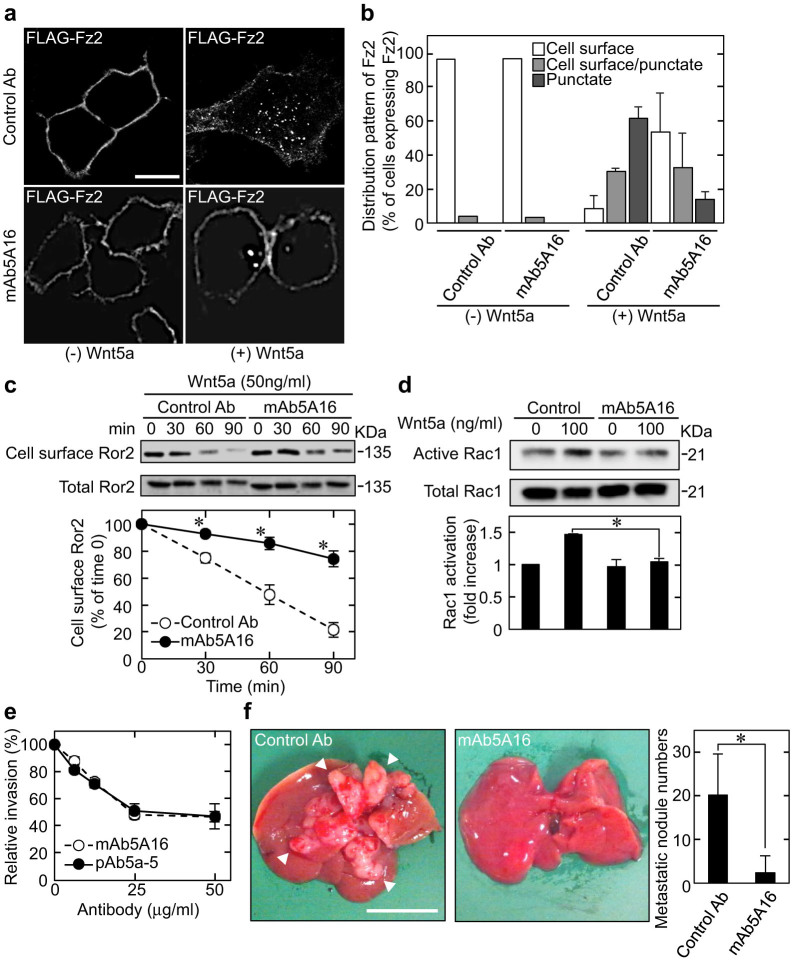
mAb5A16 inhibits KKLS cancer cell migration and invasion. (a and b) KKLS cells expressing FLAG-Fz2 were treated with 50 ng/ml Wnt5a for 30 min in the presence of 25 μg/ml anti-GST antibody (control Ab) or mAb5A16. The representative confocal images (a) and quantification of internalized FLAG-Fz2 (b) are shown. Scale bar, 10 μm.(c) KKLS cells were treated with 50 ng/ml Wnt5a for the indicated periods of time in the presence of control Ab or mAb5A16. After cell surface biotinylation, lysates were precipitated with the NeutrAvidin Agarose Resin. Top panels, the precipitates (cell surface Ror2) and lysates (total Ror2) were probed with anti-Ror2 antibody. Bottom panel, the amounts of cell surface Ror2 were quantified. Values at zero time in the presence of control Ab were set to 100%. (d) KKLS cells were stimulated with 100 ng/ml Wnt5a for 60 min in the presence or absence of mAb5A16. Cell lysates were subjected to the Rac assay. Top panels, the precipitates (active Rac1) and total lysates (total Rac1) were probed with anti-Rac1 antibody. Bottom panel, band intensities were quantified. (e) After KKLS cells were treated with the indicated concentrations of mAb5A16 or pAb5a-5, the cells were subjected to the invasion assay. Relative invasion activity was expressed as the percentage of the number of cells that invaded in the absence of the antibodies. (f) KKLS cells were injected into the spleen of nude mice in the presence of control Ab or mAb5A16. Left panels, representative results show liver metastases. Right panel, the number of metastatic liver tumors was enumerated. Scale bar, 1 cm. Results are shown as the mean ± SE of three independent experiments (b–e) and the mean ± SD of three independent samples (f). *, *P* < 0.05. Cropped blots are used. Full scan images of immunoblots are presented in Supplementary Figure S8. All the gels were run under the same experimental condition as detailed in the Methods section.

We previously demonstrated that pAb5a-5 inhibits Wnt5a-induced receptor endocytosis^22^ and that receptor-mediated endocytosis is required for Wnt5a-induced Rac1 activation and invasion ability^16^. In an immunocytochemical study of endocytosis, FLAG-Fz2 was localized to the cell surface membrane of KKLS cells in the absence of Wnt5a (Figures 2a and b). Wnt5a induced the internalization of FLAG-Fz2 after 30 min stimulation as the majority of cell surface localized FLAG-Fz2 disappeared (Figures 2a and b). However, after treatment with mAb5A16, Wnt5a-dependent internalization of FLAG-Fz2 was suppressed (Figures 2a and b). A cell surface biotinylation assay revealed that Wnt5a induced the internalization of endogenous Ror2 in a time-dependent manner, and mAb5A16 inhibited Wnt5a-induced Ror2 internalization (Figure 2c). Consistent with these findings, mAb5A16 inhibited Wnt5a-dependent Rac1 activation (Figure 2d) as well as the *in vitro* invasion capability of KKLS cells with similar efficiency to pAb5a-5 (Figure 2e). KKLS cells, a highly metastatic human gastric cancer cell line, injected into the spleen of nude mice resulted in liver metastases, which were suppressed by intraperitoneal injection of mAb5A16 (Figure 2f). However, mAb5A16 did not inhibit the interaction between Wnt5a and the extracellular cysteine rich domain of Fz2 (Fz2CRD) *in vitro* under the conditions that secreted frizzled-related protein2 (sFRP2) suppressed their interaction^28^ (Supplementary Figure S1D), indicating that mAb5A16 did not affect the binding of Wnt5a to cell surface receptors but instead inhibited Wnt5a-induced receptor internalization. These results indicate that our newly generated mAb5A16 suppresses Wnt5a-dependent internalization of receptors, resulting in the prevention of gastric cancer cell metastasis by inhibiting the Rac1 activity.

### Wnt5a expression promotes cancer cell proliferation

**Figure 3:**
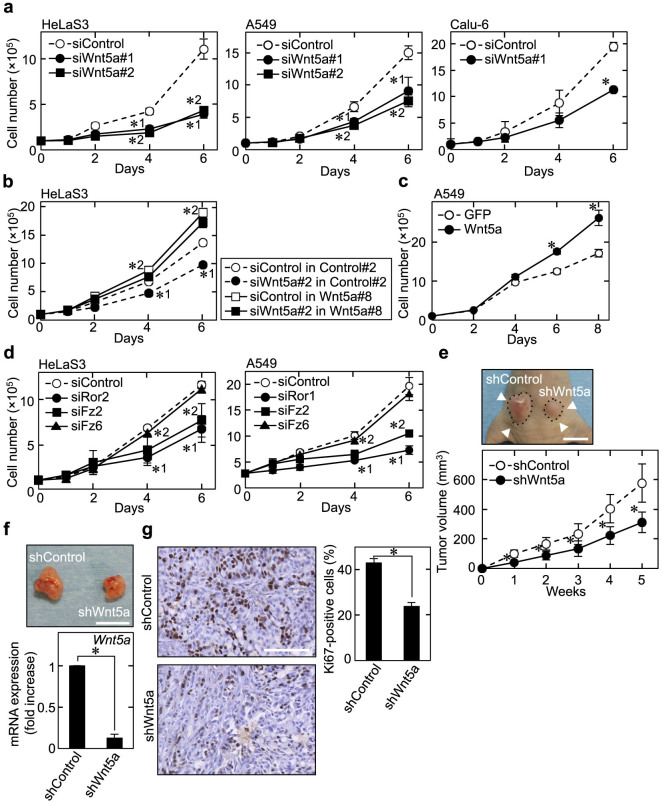
Wnt5a stimulates proliferation of HeLaS3 and A549 cells. (a) HeLaS3 (left panel), A549 (middle panel), and Calu-6 (right panel) cells transfected with the indicated siRNAs were subjected to the cell proliferation assay. *1 and *2 indicate *P* < 0.05 between siControl and siWnt5a#1, and between siControl and siWnt5a#2, respectively. (b) HeLaS3 cells stably expressing the neomycin resistance gene (Control#2) or Wnt5a (Wnt5a#8) were transfected with control siRNA (siControl) or *Wnt5a* siRNA#2 (siWnt5a#2), and cells were then subjected to the cell proliferation assay. *1 and *2 indicate *P* < 0.05 between siControl and siWnt5a#2 in Control#2, and between siControl in Control#2 and siControl in Wnt5a#8, respectively. (c) A549 cells stably expressing GFP or Wnt5a were subjected to the proliferation assay. (d) HeLaS3 or A549 cells transfected with the indicated siRNAs were subjected to the proliferation assay. *1 and *2 indicate *P* < 0.05 between siControl and siRor2, and between siControl and siFz2, respectively.(e) A549 cells (5 × 10^6^ cells) stably expressing control shRNA (shControl) or Wnt5a shRNA (shWnt5a) were subcutaneously implanted into nude mice for 5 weeks (n = 4). Top panel, dashed lines show outline of xenografts, and arrowheads indicate positions of tumors. Bottom panel, the tumor volumes were plotted every week. Scale bar, 1 cm. (f) Top panel, xenograft tumors were isolated from nude mice in Figure 3e. Bottom panel, *Wnt5a* mRNA levels in xenografts were examined by semi-quantitative RT-PCR analysis. Scale bar, 1cm. (g) Left panels, sections prepared from xenografts were stained with hematoxylin and anti-Ki-67 antibody (brown color). Right panel, Ki-67-positive cells were counted and the results are expressed as the percentage of positively-stained cells compared to the total number of cells per field. Scale bar, 100 μm. Results are shown as the mean ± SE of three independent experiments (a–d, f, and g) and the mean ± SD of four independent experiments (e). *, *P* < 0.05.

In addition to the role of Wnt5a in cell motility via cytoskeletal regulation, Wnt5a has recently been demonstrated to be involved in cell proliferation^19^,^20^,^21^. *Wnt5a* and Wnt receptor mRNA levels varied among cancer cell lines (Supplementary Figure S2A). Knockdown of Wnt5a (Supplementary Figures S2B–D) indeed suppressed proliferation of HeLaS3 cervical cancer cells as well as A549 and Calu-6 lung cancer cells (Figure 3a). Stable expression of Wnt5a (Wnt5a#8) promoted proliferation of HeLaS3 cells (Figure 3b and Supplementary Figure S2E) and rescued inhibited proliferation by depletion of Wnt5a (Figure 3b and Supplementary Figure S2F). Another Wnt5a expression clone of HeLaS3 cells (Wnt5a#9) also showed enhanced cell proliferation and did not decrease the proliferation when Wnt5a was depleted (data not shown). In addition, Wnt5a expression promoted A549 cell proliferation (Figure 3c and Supplementary Figure S2G). Knockdown of Fz2, Ror1, or Ror2 in HeLaS3 and A549 cells (Supplementary Figures S2H and I) inhibited proliferation, but Fz6 knockdown did not (Figure 3d). Cell migration and invasion were also suppressed in Wnt5a-, Ror1-, Ror2-, or Fz2-depleted HeLaS3 and A549 cells (Supplementary Figure S3), suggesting that both cell proliferation and migration capabilities of these cancer cells depend on Wnt5a signaling through its receptors. The role of Wnt5a expression in tumorigenesis *in vivo* was investigated by subcutaneously implanting A549 cells into the flanks of nude mice. Xenograft tumor formation was reduced by Wnt5a depletion (Figures 3e and f), and Ki67 (a cell proliferation marker) expression levels were also reduced in Wnt5a-depleted tumor cells (Figure 3g).

Proliferation of KYSE-70 and TE-11 esophageal cancer cells, in which Wnt5a was expressed at higher levels than in HeLaS3 cells (Supplementary Figure S4A), was also suppressed by knockdown of Wnt5a (Supplementary Figures S4B and C). Taken together, these results indicate that Wnt5a promotes proliferation of certain types of cancer cells in which endogenous Wnt5a is highly expressed.

### Blockade of receptor-mediated endocytosis does not affect Wnt5a-induced proliferation of cancer cells

**Figure 4:**
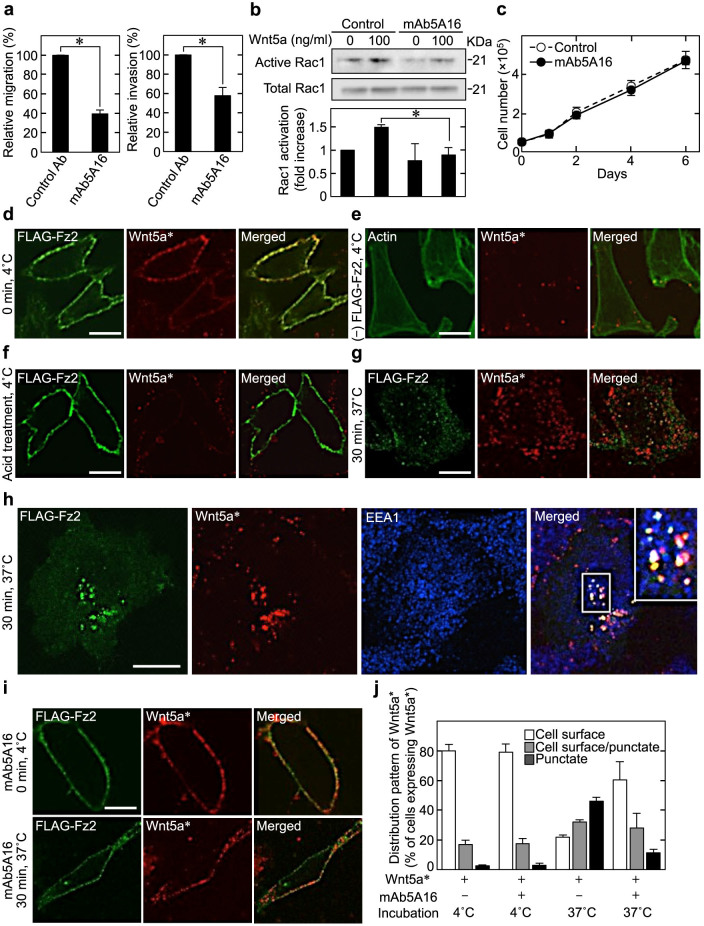
Receptor-mediated endocytosis is not involved in Wnt5a-induced cell proliferation. (a) HeLaS3 cells treated with 25 μg/ml control Ab or mAb5A16 were subjected to the migration and invasion assays. (b) HeLaS3 cells were stimulated with 100 ng/ml Wnt5a for 60 min in the presence or absence of mAb5A16; cell lysates were subjected to the Rac assay. (c) HeLaS3 cells treated with control Ab or mAb5A16 were subjected to the proliferation assay. (d and e) HeLaS3 cells expressing (d) or not expressing (e) FLAG-Fz2 were incubated with 500 ng/ml Wnt5a* (labelled Wnt5a) at 4°C. FLAG-Fz2 is shown in green and Wnt5a* is in red. In the merged images, co-localization of FLAG-Fz2 and Wnt5a* appears yellow. (f) HeLaS3 cells expressing FLAG-Fz2 were incubated with Wnt5a* at 4°C; cells were then washed three times with an acid solution (100 mM NaCl and 100 mM glycine/HCl [pH 2.8]) to remove Wnt5a* bound to the receptors at the cell surface. (g and h) HeLaS3 cells expressing FLAG-Fz2 were incubated with Wnt5a* at 4°C; then the cells were incubated for 30 min at 37°C. The cells were stained with anti-FLAG (g and h) and anti-EEA1 (h) antibodies. Co-localization of FLAG-Fz2, EEA1, and Wnt5a* appears white. (i and j) HeLaS3 cells expressing FLAG-Fz2 were treated with Wnt5a* for 30 min in the presence of mAb5A16. FLAG-Fz2 is shown in green and Wnt5a* is in red. Co-localization of FLAG-Fz2 and Wnt5a* appears yellow. The representative confocal images (i) and quantification of internalized Wnt5a* (j) are shown. Results are shown as the mean ± SE of three independent experiments. Scale bars, 10 μm. *, *P* < 0.05. Cropped blots are used. Full scan images of immunoblots are presented in Supplementary Figure S9. All the gels were run under the same experimental condition as detailed in the Methods section.

In HeLaS3 cells, mAb5A16 inhibited migration, invasion, and Wnt5a-dependent activation of Rac1 (Figures 4a and b), but not cell proliferation (Figure 4c). It did not affect proliferation of A549 cells, either (data not shown). The effect of mAb5A16 on Wnt5a-induced receptor internalization in cancer cells which proliferate in a Wnt5a-dependent manner was examined. Purified Wnt5a was labelled with AlexaFluor 546 carboxylic acid succinimidyl ester to directly detect the internalization of Wnt5a. AlexaFluor 546 was conjugated to Wnt5a at a molar ratio of 3.5:1 (Supplementary Figure S5A). Although the specific activity of labelled Wnt5a (Wnt5a*) that induces the phosphorylation of Dvl2 in NIH3T3 cells was partially reduced (Supplementary Figure S5A), we used this labelling condition in the following assays because the labelling of Wnt5a with AlexaFluor 546 at a higher molar ratio (5.5-6:1) resulted in the complete loss of Wnt5a activity (Supplementary Figure S5B).

Wnt5a* was observed on HeLaS3 cell surface membranes at 4°C when FLAG-Fz2 was expressed (Figure 4d), while Wnt5a* was not observed when FLAG-Fz2 was not expressed (Figure 4e). Wnt5a* was removed from the cells by washing with an acid solution (Figure 4f), suggesting that Wnt5a* binds to the cell surface receptor. At 30 min after the temperature was shifted to 37°C, Wnt5a* disappeared from cell surface membranes and was observed as punctate structures in the cytoplasm (Figures 4g and h). Internalized Wnt5a* was co-localized with more than 80% of FLAG-Fz2 (Figures 4g and h, and Supplementary Figure S5C); the complex was partially co-localized with EEA1 (Figure 4h), suggesting that Wnt5a* was internalized with FLAG-Fz2 and trafficked to the early endosomes. These results show that Wnt5a* can be used as a new tool to analyze the intracellular fate of Wnt5a after receptor binding. In the presence of mAb5A16, Wnt5a* was located to the cell surface at 4°C and most of Wnt5a* was not internalized with FLAG-Fz2 after the cells were incubated at 37°C (Figures 4i and j).

**Figure 5:**
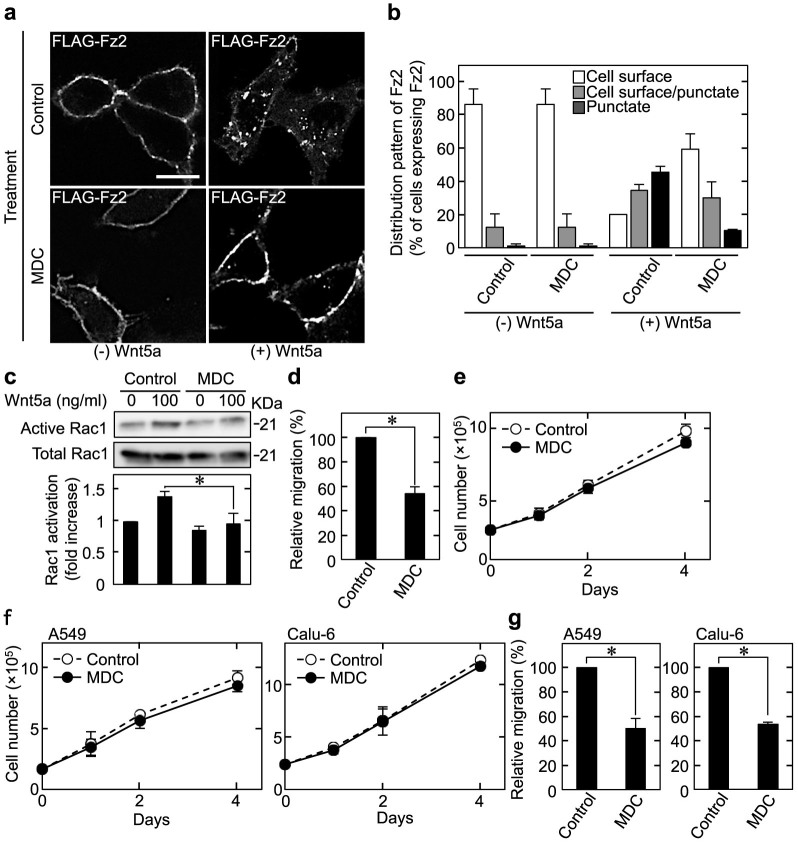
Clathrin-mediated endocytosis is not involved in Wnt5a-induced cell proliferation. (a and b) HeLaS3 cells expressing FLAG-Fz2 were pre-treated with 7.5 μM MDC for 48 h; then the cells were incubated with 100 ng/ml Wnt5a for 30 min. The representative confocal images (a) and quantification of internalized FLAG-Fz2 (b) are shown. Scale bar, 10 μm. (c) HeLaS3 cells were stimulated with Wnt5a for 60 min after treatment with MDC for 48 h, and then cell lysates were subjected to the Rac assay. (d and e) HeLaS3 cells were treated with or without MDC; then cells were subjected to the migration (d) and proliferation (e) assays. (f and g) A549 (left panel) and Calu-6 (right panel) cells were treated with or without MDC; then cells were subjected to the proliferation (f) and migration (g) assays. Results are shown as the mean ± SE of three independent experiments. *, *P* < 0.05. Cropped blots are used. Full scan images of immunoblots are presented in Supplementary Figures S10. All the gels were run under the same experimental condition as detailed in the Methods section.

Monodansylcadavelin (MDC) promotes the assembly of clathrin, resulting in the inhibition of clathrin-dependent receptor endocytosis^29^. Under previous assay conditions, treatment of cells with 50 μM MDC for 30 min inhibited Wnt5a-induced Fz2 internalization and Rac1 activation^16^. Cells have to be treated with MDC for a long time to examine the involvement of endocytosis in cell proliferation. To avoid the cytotoxic effects induced by long-term treatment with MDC, its concentration was reduced. When HeLaS3 cells were treated with 7.5 μM MDC for 48 h, cell death was not observed as indicated using trypan blue staining assays (data not shown). Treatment of HeLaS3 cells with 7.5 μM MDC for 48 h inhibited Wnt5a-induced Fz2 internalization (Figures 5a and b) and Rac1 activation (Figure 5c). These observations were confirmed using HeLaS3 cells stably expressing Wnt5a (Wnt5a#8). Fz2 internalization was promoted in Wnt5a#8 cells as compared with control HeLaS3 cells (Control#2), and mAb5A16 and MDC inhibited Fz2 internalization in Wnt5a#8 cells (Supplementary Figure S6). In addition, MDC suppressed migration (Figure 5d) but not affected proliferation (Figure 5e) of HeLaS3 cells.

As well as HeLaS3 cells, MDC did not inhibit proliferation of A549 and Calu-6 cells (Figure 5f) under the conditions that MDC suppressed migration activity of these cancer cells (Figure 5g). Taken together, these results suggest that Wnt5a-dependent cancer cell proliferation is regulated by a receptor-mediated endocytosis-independent mechanism.

### Wnt5a activated Src family kinases in a receptor-mediated endocytosis-independent manner

**Figure 6:**
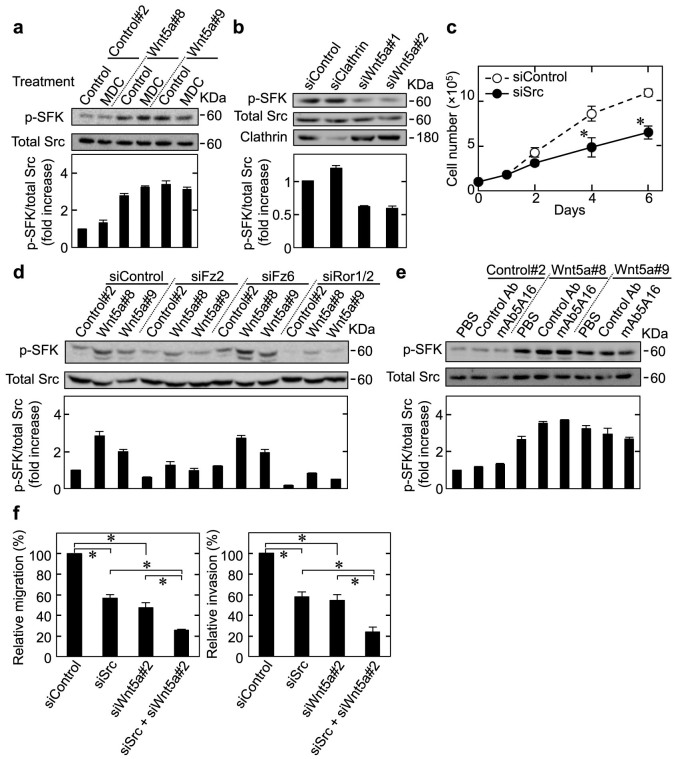
Wnt5a-induced proliferation of HeLaS3 cells depends on SFKs in a receptor-mediated endocytosis-independent manner. (a) Top panels, HeLaS3 cells stably expressing the neomycin resistance gene (Control#2) or Wnt5a (Wnt5a#8 or #9) were treated with or without MDC for 48 h, and lysates were then probed with the indicated antibodies. Activated SFKs that are phosphorylated at the position of Tyr416 indicates p-SFK. Bottom panel, band intensities of p-SFK at the position of Tyr416 were normalized with band intensities of total Src in each lane. Results are shown as the fold increase compared with the intensity in control cells. (b) HeLaS3 cells were transfected with the indicated siRNAs, and lysates were then probed with the indicated antibodies. (c) HeLaS3 cells were transfected with control or *Src* siRNA, and then subjected to the proliferation assay. (d) Control#2, Wnt5a#8, or Wnt5a #9 cells were transfected with the indicated siRNAs for 48 h, and lysates were then probed with the indicated antibodies. (e) Control#2, Wnt5a#8, or Wnt5a#9 cells were treated with 25 μg/ml control Ab or mAb5A16 for 48 h, and lysates were then probed with the indicated antibodies. (f) HeLaS3 cells transfected with the indicated siRNAs were subjected to the migration (left panel) and invasion (right panel) assays. Relative migration and invasion activities were expressed as percentages of those in control cells. Results are shown as the mean ± SE of three independent experiments. *, *P* < 0.05. Cropped blots are used. Full scan images of immunoblots are presented in Supplementary Figures S11 and S12. All the gels were run under the same experimental condition as detailed in the Methods section.

To understand the mechanism underlying Wnt5a-dependent cell proliferation, we analyzed several intracellular signaling molecule activities, including Src family kinases (SFKs), AKT, PKC, and JNK in HeLaS3 cells. Among these signaling molecules, expression and knockdown of Wnt5a activated and inhibited, respectively, SFK activity, which was assessed by the tyrosine phosphorylation of SFK (p-SFK) (Figures 6a and b), but not other kinase activities (Supplementary Figure S7A). Src and Fyn but not Yes were expressed in HeLaS3 cells, and p-SFK indeed decreased by knockdown of Src or Fyn in HeLaS3 (Supplementary Figures S7B and C). Src depletion suppressed HeLaS3 cell proliferation (Figure 6c). Knockdown of Fz2 and Ror1/2 suppressed SFK activity, but that of Fz6 did not (Figure 6d). The treatment with MDC or clathrin knockdown did not affect Wnt5a-dependent SFK activity of HeLaS3 cells (Figures 6a and b). In addition, mAb5A16 did not affect Wnt5a-induced SFK activity (Figure 6e).

Src but not Fyn or Yes was expressed in A549 and Calu-6 cells (Supplementary Figure S7B). Thus, Src was a major component of SFKs in A549 and Calu-6 cells and the SFK activity was reduced by knockdown of Wnt5a but not clathrin in these cancer cells (Supplementary Figure S7D). As well as proliferation, basal and Wnt5a-induced SFK activation in A549 and Calu-6 cells was not suppressed by MDC (Supplementary Figure S7E). These results suggest that Wnt5a-dependent SFK activation is involved in cell proliferation, but does not require receptor-mediated endocytosis.

Src depletion also inhibited the migration and invasion capabilities of HeLaS3 cells to the similar levels to Wnt5a depletion, and knockdown of both Wnt5a and Src further suppressed migration and invasion (Figures 6f). Taken together with the observations that receptor-mediated endocytosis is involved in Wnt5a-dependent migration and invasion, these results suggest that Wnt5a and Src might regulate these cellular functions independently.

## Discussion

In this study we generated an anti-Wnt5a monoclonal antibody (mAb5A16) from a phage library; this antibody inhibited invasion and metastasis of gastric cancer cells both *in vitro* and *in vivo*. Epitope mapping analyses revealed that mAb5A16 recognizes 211–217 amino acids of Wnt5a. Judging from the three dimensional structure of XWnt8^27^, this region is unlikely to be involved in the binding of Wnt5a to Fz, because Wnt5a was predicted to bind to Fz through two regions that are distinct from the region recognized by mAb5A16. Consistent with these observations, mAb5A16 did not affect the binding of Wnt5a to Fz2. It was reported that Wnt3a recognizes LRP6 through the 241–263 amino acids, including acidic amino acid region^30^. Wnt5a has a similar acidic region that might be involved in the binding to Ror2, but the acidic region is also different from the mAb5A16 recognition region, suggesting that mAb5A16 fails to affect the binding of Wnt5a to Ror2. The region recognized by mAb5A16 was spatially close to the epitope for pAb5a-5. Similar to pAb5a-5, mAb5A16 inhibited Wnt5a-induced receptor-mediated endocytosis. Therefore, the Wnt5a regions recognized by these two antibodies could be important for Wnt5a-activated signal transduction through the receptor complex internalization.

It has been reported that glypican-4 (GPC4) overexpression in HeLaS3 cells promotes Wnt5a-induced receptor-mediated endocytosis and Rac1 activation and that Wnt5a induces the internalization of GPC4 with Fz2, followed by the localization of the complex to clathrin-positive vesicles^31^. Heparan sulfate proteoglycans are important for Wnt signaling and associated with developmental processes^3^,^32^. Thus, it is intriguing to speculate that the three-dimensional structure of Wnt5a, encompassing epitopes for both mAb5A16 and pAb5a-5, binds to a third factor such as GPC4, which then plays a role in Wnt5a-induced receptor-mediated endocytosis.

As a new tool to analyze Wnt5a signaling, we labelled purified Wnt5a protein using a fluorescent probe. Labelled Wnt5a (Wnt5a*) specifically bound to cell surface Fz2 and caused the internalization of Fz2. We were not able to rule out that Wnt5a* used in our experiments may be a mixture of labelled and unlabelled Wnt5a. However, because most of Wnt5a* was internalized in the presence of FLAG-Fz2 and internalized Wnt5a* was co-localized with more than 80% of FLAG-Fz2, a large part of Wnt5a could be labelled and keep the activity. Therefore, Wnt5a* is a useful tool to monitor the trafficking route of Wnt as it travels from the cell surface to intracellular destination. Using this method, the ability of mAb5A16 to inhibit Wnt5a-induced receptor internalization without affecting the binding of Wnt5a to its receptors was confirmed. Endogenous Wingless, the *Drosophila* Wnt homologue, and endogenous Wnt5a can be detected by their antibodies in imaginal discs and mammalian cells, respectively^33^,^34^. An ectopically expressed Wnt-EGFP fusion protein was detected to be associated with microtubules in *Xenopus* tissue culture cells^35^. However, in these assays, it is hard to examine the trafficking route of Wnt and receptors. Obtaining fluorescently-tagged active Wnt proteins is challenging because covalent modification of fluorescent dyes interferes with Wnt activity. We overcame these difficulties by using our purified Wnt5a protein that has a high specific activity. Minimizing the loss of Wnt activity and enhancing the sensitivity of probe could make this method an even more powerful tool for the analysis of Wnt signaling.

Wnt5a induces clathrin-dependent receptor-mediated endocytosis, resulting in the activation of the β-catenin-independent pathway^16^,^36^, whereas Wnt3a induces caveolin-dependent receptor-mediated endocytosis, resulting in the activation of the β-catenin-dependent pathway^6^,^37^,^38^,^39^. However, the present study suggests that the endocytosis-independent mechanism exists in the β-catenin-independent pathway to regulate Wnt5a-induecd cancer cell proliferation. Although it has been shown that the Wnt5a signal is not involved in the proliferation of gastric and prostate cancer cells^11^,^14^, Wnt5a did promote the proliferation of cervical, lung, and esophageal cancer cells. In the latter types of cancer cells, impairment of Wnt5a-induced receptor-mediated endocytosis by mAb5A16 or MDC did not affect cell proliferation. Although the entire mechanism by which Wnt5a stimulates cancer cell proliferation is still not fully understood, SFKs might be involved in this pathway. These experiments were done using HeLaS3 cells stably expressing Wnt5a. However, when HeLaS3 cells were stimulated with purified Wnt5a protein, SFK activation and cell proliferation were not observed (data not shown). Overexpression and knockdown of Wnt5a stimulates and inhibits, respectively, the SFK activity and cell proliferation in HeLaS3 and A549 cells. Therefore, other factors released from the cells might cooperate with Wnt5a to stimulate these cellular events. It has been reported that fluid phase and receptor-mediated endocytosis are inhibited during mitosis^40^, implicating that the endocytic process is not required for the control of mitotic phase. Taken together with the finding that Wnt5a-dependent cell proliferation does not require receptor internalization, it is intriguing to speculate that Wnt5a signaling promotes mitotic phase progression to stimulate cell proliferation in a receptor endocytosis-independent manner.

SFKs are localized to the inner surface of the plasma membrane and primarily transmits signals downstream of receptor tyrosine kinases (RTKs) and integrins to regulate cell proliferation, motility, and survival^41^,^42^,^43^. Wnt5a-dependent Src activation occurs in osteosarcoma cells^44^, Ror2 is associated with and activates Src in melanoma cells^45^, and Src phosphorylation of Ror2 leads to Ror2 internalization in Rab5 positive endosomes^46^. The Wnt5a and Fz2 complex associates with integrin α2 to regulate focal adhesion dynamics in migrating cells^47^. In addition it has been recently reported that Fyn mediates Wnt5a/b- and Fz2-induced epithelial-mesenchymal transition and tumor metastasis^48^. Thus, SFKs are involved in the regulation of cell motility in the context of Wnt5a signaling. This study revealed that augmenting Wnt5a signaling activated Src and Fyn in HeLaS3 cells and promoted cell proliferation, but inhibition of clathrin-dependent receptor internalization did not affect the SFK activity. Therefore, Wnt5a might activate SFKs in a receptor-mediated endocytosis-independent manner, thereby stimulating proliferation. In contrast, taken together with the observations that Wnt5a-dependent migration and invasion require receptor-mediated endocytosis, Src might regulate cell migration and invasion of HeLaS3 cells in a Wnt5a-independent manner. This possibility is likely, because Src and its family are involved in the regulation of focal adhesion^49^,^50^.

mAb5A16 inhibited the invasion capability of KKLS and HeLaS3 cells to the 50–55% level (this study), and pAb5a-5 also suppressed the invasion ability of KKLS and MKN-1 cells to the 40~50% level^22^. Taken together with the observations that knockdown of Wnt5a reduces the invasion activity of these cancer cells to the half level, these results suggest that the signaling pathway(s) activated by molecules other than Wnt5a are involved in these cell invasion.

In the case of the β-catenin-dependent pathway, ring finger 43 (RNF43)/zinc and ring finger 3 (ZNRF3), a transmembrane E3 ligase, ubiquitinates and reduces cell surface Fz associated with LRP6, thereby suppressing intracellular signaling^51^,^52^. In the presence of R-spondin, it binds to both RNF43/ZNRF3 and leucine-rich repeat containing G protein-coupled receptor (LGR) 4/5/6 and removes RNF43/ZNRF3 from Fz, followed by the internalization of RNF43/ZNRF3 and LGR4/5/6. As a result, R-spondin acts to keep Fz on the cell surface and enhances the β-catenin-dependent signal^53^,^54^. Thus, R-spondin seems to switch and tune the signal intensity of the β-catenin-dependent pathway on cell surface by preventing the receptor internalization. Therefore, unidentified extracellular factors might activate the β-catenin-independent pathway in cooperation with Wnt5a without the receptor internalization.

In summary, we found that Wnt5a signal regulates invasion and proliferation of cancer cells through receptor-mediated endocytosis-dependent and -independent mechanisms, respectively, using our newly generated anti-Wnt5a neutralizing monoclonal antibody and labelled Wnt5a protein.

## Methods

### Materials and chemicals

Standard recombinant DNA techniques were used to construct pCS2/FLAG-rat Fz2 (FLAG-Fz2) and pPGK-neo/Wnt5a. pSuper-retro-GFP-Neo-shWnt5a, which was used for the generation of cells stably expressing Wnt5a shRNA, was generated by inserting small hairpin RNA against Wnt5a (5′-GTGGATAACACCTCTGTT-3′) into the pSuper-retro-GFP-Neo vector (Oligo Engine, Seattle, WA). The small interfering RNAs (siRNAs) used in this study are listed in Table S1. Wnt5a was purified to near homogeneity using three chromatography steps as described previously^16^,^55^. Anti-Wnt3a, anti-Wnt5a/b, anti-Src, anti-Fyn, anti-Yes, anti-phospho-Src family (Tyr416), anti-AKT, anti-phospho-AKT (Ser473), anti-phospho-PKC (pan) (Ser660), anti-SAPK/JNK, and anti-phospho-SAPK/JNK (Thr183/Tyr185) antibodies were purchased from Cell Signaling Technology (Beverly, MA). Anti-Rac1, anti-EEA1, anti-HSP90, anti-PKCα, and anti-Clathrin antibodies were from BD Biosciences (San Jose, CA). Anti-FLAG M2 and anti-Ror2 antibodies were from Sigma-Aldrich (Steinheim, Germany) and R&D Systems (Minneapolis, MN), respectively.

### Generation of anti-Wnt5a monoclonal antibody from the HuCAL Platinum Library

Human Wnt5a monoclonal antibodies were developed in Shionogi & Co., Ltd. The HuCAL Platinum antibody library (MorphoSys, Munic, Germany) was used for the selection of anti-Wnt5a antibodies. Anti-Wnt5a antibodies were identified by recombinant protein-based panning according to the manufacturer's instructions. His-Wnt5a, which was expressed in and purified from *E. coli*, was captured on Maxisorp plates (Thermo Scientific, Waltham, MA) and incubated with pools of HuCAL Platinum phage. After three rounds of selection, the enriched antibody DNA in phagemids were excised as a pool and cloned onto the expression vector pMORPHX9_Fab_FH. Fab fragments expressed from each clones were screened on His-Wnt5a-coated Maxisorp plates. Fab fragments with affinity for Wnt5a were purified and evaluated in transwell migration and invasion assays. Conversion into IgG was performed by sub-cloning variable domain fragments of heavy and light chains from Fab expression vectors into the pMORPH2_human/rat_Ig vector series to generate the anti-Wnt5a monoclonal antibody, mAb5A16. The DNA for mAb5A16 was expressed in HEK293 cells and the monoclonal antibody was subsequently purified using protein A-Sepharose.

### Epitope prediction

The epitopes recognized by mAb5A16 and pAb5a-5 were predicted using a pepspot analysis according to the manufacturer's instructions (JPT Peptide Technologies, Berlin, Germany). Pepspot membrane, including of 38 peptides, each 17-amino acids in length with 7-amino acids overlapping adjacent peptides, was probed with 2 μg/ml antibody each. Bound antibodies were detected using horseradish peroxidase (HRP)-conjugated secondary antibodies and ECL™ Western blotting detection reagents (GE healthcare, Buckinghamshire, UK). Signals were detected using X-ray film (GE healthcare). All structural figures were prepared using the PyMOL Molecular Graphics System (Version 1.3r1)^56^.

### Cell cultures

HeLaS3, A549, KYSE-70, TE-11, Calu-6, and NIH3T3 cells were grown in Dulbecco's modified Eagle's medium (DMEM) supplemented with 10% fetal bovine serum (FBS). KKLS and MKN-45 cells were grown in RPMI-1640 supplemented with 10% FBS. To generate HeLaS3 or MKN-45 cells stably expressing Wnt5a, HeLaS3 or MKN-45 cells were transfected with pPGK-neo/Wnt5a and selected with 400 μg/ml G418; colonies of resistant cells were isolated. Control#2 and two different HeLaS3 cell lines expressing Wnt5a (Wnt5a#8 and #9) were used in this study. To generate A549 cells stably expressing either GFP or Wnt5a, 5 × 10^4^ parental cells/well in a 12-well plate were infected with lentiviruses expressing GFP or Wnt5a. Cells were selected and maintained in medium containing 800 μg/ml G418. To generate A549 cells stably expressing Wnt5a shRNA, A549 cells were transfected with pSuper-retro-GFP-Neo-shWnt5a and selected with 800 μg/ml G418; colonies of resistant cells were isolated. The Wnt5a-depleted cells were used in xenograft tumor assays. When necessary, the cells were treated with 7.5 μM monodansylcadaverine (MDC).

### Labelling of Wnt5a

Purified Wnt5a (40 pmol) was fluorescently labelled with AlexaFluor 546 carboxylic acid succinimidyl ester (200 pmol; Life Technologies, Carlsbad, CA) in 200 mM sodium hydrogen carbonate [pH 7.4] for 3 h at 4°C. After quenching of excess AlexaFluor 546 with 300 mM hydroxylamine, labelled Wnt5a (Wnt5a*) was used for a receptor internalization assay. The degree of labelling of Wnt5a with AlexaFluor 546 was estimated by comparison to AlexaFluor 546-conjugated streptavidin (4 mol of AlexaFluor 546 conjugated to 1 mol of streptavidin, Life Technologies) using a fluorescence image analyzer (Tyhoon, GE). About 3.5 mol of AlexaFluor 546 was conjugated to 1 mol Wnt5a and Wnt5a* was used for the internalization assay.

### Internalization of FLAG-Fz2 and Wnt5a*

The ligand and receptor internalization assay was performed as described previously^6^,^16^. Briefly, after KKLS, HeLaS3, or HeLaS3 cells stably expressing Wnt5a (Wnt5a#8) cells were transfected with pCS2/FLAG-Fz2, the cells were incubated with ice-cold binding medium (RPMI-1640 or DMEM containing 20 mM Hepes-NaOH [pH 7.5] and 0.1% bovine serum albumin (BSA)) for 30 min and further incubated in the presence of 500 ng/ml anti-FLAG antibody with 25 μg/ml anti-glutathione-*S*-transferase (GST) antibody (control Ab) or mAb5A16 for 60 min at 4°C. When necessary, the cells were treated with 100 ng/ml Wnt5a or 500 ng/ml Wnt5a* at this time. After unbound Wnt5a, Wnt5a* and antibodies were removed by washing with cold PBS three times, internalization was initiated by adding warm (37°C) RPMI-1640 (KKLS), DMEM (HeLaS3), or DMEM supplemented with 10% FBS (Wnt5a#8) and the dishes were transferred to a heated chamber (37°C, 5% CO_2_) for 30 or 60 min. After the cells were washed three times with cold PBS to stop endocytosis, the cells were fixed, they were probed with AlexaFluor 488-conjugated anti-mouse IgG (Life Technologies) and viewed using a confocal microscope (LSM510, Carl Zeiss, Jana, Germany) to observe FLAG-Fz2 or Wnt5a*. To quantify the distribution of FLAG-Fz2 or Wnt5a*, their localization was classified into three types as described^6^,^16^. More than 100 cells were evaluated in each experiment.

### Cell proliferation assay

HeLaS3, A549, Calu-6, KYSE-70, or TE-11 cells were seeded at densities of 1 × 10^5^/ml. HeLaS3 cells were cultured with 10% FBS while the other cells were cultured with 5% FBS. Cells were enumerated on the indicated days.

### Generation of lentiviruses

Wnt5a and EGFP cDNAs were cloned into the pLVSIN-EF1α Neo Vector (Takara Bio Inc., Shiga, Japan) to construct lentivirus vectors. Lentiviruses were produced in X293T cells by using the Lenti-XTM Lentiviral Expression Systems (Takara Bio Inc.) in accordance with the manufacturer's instructions.

### Enzyme-linked immunosorbent (ELISA)-based affinity assay

For the assessment of the affinity of mAb5A16 or pAb5a-5 for Wnt5a, a 96-well plate was coated with 25 μl of 2 μg/ml each antibody at 4°C overnight; the plate was then blocked with PBS containing 1% BSA for 2 h at room temperature. After blocking, 25 μl of the indicated concentrations of purified Wnt5a were added for 2 h at room temperature, washed with PBS containing 0.05% Tween-20 three times, and then detected using biotinylated monoclonal anti-Wnt5a antibody (R&D Systems).

For the assessment of the ability of mAb5A16 to affect the binding of Wnt5a and Fz2, a 384 well plate was coated with 25 μl of 2 μg/ml control-IgG or 25 μl of 1 μg/ml purified Fz2CRD-IgG at 4°C overnight. The plate was blocked with PBS containing 1% BSA for 2 h at room temperature. After blocking, 25 μl of 500 ng/ml Wnt5a were added with 25 μl of 250 μg/ml anti-GST antibody (control Ab) or mAb5A16 or 25 μl of 10 μg/ml of purified sFRP2 for 2 h at room temperature, washed with PBS containing 0.05% Tween-20 three times, and then detected using biotinylated monoclonal anti-Wnt5a antibody (R&D Systems).

### Xenograft tumor assay

The protocols used for all animal experiments in this study were approved by the Animal Research Committee of Osaka University, Japan (No. 21-048-1). All animal experiments were carried out according to the guidelines for the care and use of experimental animals of Osaka University. The *in vivo* tumor metastasis assay was performed as described^22^ except that mAb5A16 was used. For the *in vivo* tumor formation assay, A549 (5 × 10^6^) cells expressing control or Wnt5a shRNA in 100 μl of PBS were injected subcutaneously into the dorsal surfaces of nude mice through a 27-gauge needle (day 0). Five weeks after transplantation, the mice were sacrificed and the transplanted samples were measured, weighed, and processed for histological analysis.

### Semi-quantitative RT-PCR

Semi-quantitative RT-PCR was performed as described previously^57^. Forward and reverse primers are listed in Table S2.

### Statistical analysis

The experiments were performed at least three times and results were expressed as the mean ± standard deviation (SD) or the mean ± standard error (SE). Differences between control and experimental groups were evaluated using the Student's *t*-test. A *P* value < 0.05 was considered a significant difference.

### Others

Ror2 internalization at endogenous levels using cell surface biotinylation was examined as described previously^22^. Rac activity was assayed using GST-CRIB as described^16^,^58^. Quantification data was calculated based on at least three blots from different experiments. Migration capabilities of KKLS, HeLaS3, and A549 cells were measured using a modified Boyden chamber^10^,^14^. When necessary, 25 μg/ml mAb5A16, pAb5a-5, or anti-GST antibody were used. The invasive potential of the cells was analyzed using a matrigel-coated modified Boyden chamber (BD Biosciences) as described previously^10^,^11^.

## Author Contributions

K.S. designed the experiments, carried out the mouse and cell level experiments, and wrote the manuscript. A.S. designed experiments, carried out the cell level experiments, and wrote the manuscript. H.H., I.T. and H.Y. carried out the mouse and cell level experiments. M.N., K.H., Y.S., K.D. and M.H. generated a neutralizing anti-Wnt5a monoclonal antibody. A.K. designed experiments and wrote the manuscript.

## Supplementary Material

Supplementary Information Supplementary Info
