# Supplementary material for: Wnt5a promotes cancer cell invasion and proliferation by receptor-mediated endocytosis-dependent and -independent mechanisms, respectively
Source: Sci Rep. 2015 Jan 27;5:8042. doi: 10.1038/srep08042 (PMC4306915; doi:10.1038/srep08042)
Supplement: Supplementary Information — Supplementary Info [file srep08042-s1.pdf]

Supplementary Information

Wnt5a promotes cancer cell invasion and proliferation by receptor-mediated endocytosis-dependent and -independent mechanisms, respectively

Kensaku Shojima[1], Akira Sato[1], Hideaki Hanaki[1], Ikuko Tsujimoto[1], Masahiro Nakamura[2], Kazunari Hattori[3], Yuji Sato[4], Keiji Dohi[4], Michinari Hirata[4], Hideki Yamamoto[1], and Akira Kikuchi[1,]*

[1]Department of Molecular Biology and Biochemistry, Graduate School of Medicine, Osaka University, 2-2 Yamadaoka, Suita 565-0871, Japan. [2]Diagnostics Division, [3]Department of Informatics & Structure-based Drug Discovery, [4]Department of Oncology & Immunology, Discovery Research Laboratory for Innovative Frontier Medicines, Shionogi & Co., Ltd. 1-1, Futaba-cho 3-chome, Toyonaka, 561-0825, Japan.

*Correspondence author. Department of Molecular Biology and Biochemistry, Graduate School of Medicine, Osaka University, 2-2 Yamadaoka, Suita 565-0871, Japan.

Phone: 81-6-6879-3410. Fax: 81-6-6879-3419.

E-mail: akikuchi@molbiobc.med.osaka-u.ac.jp.

Supplementary Figure legends

Figure S1. Generation of anti-Wnt5a rat monoclonal antibody.

(A) KKLS cells treated with 25 μg/ml Fab fragments (#4, #6, #8, #14, #16, #20, #27, or #31) or pAb5a-5 were subjected to the invasion assay. Relative invasion activities were expressed as percentages of PBS-treated control cells.

(B) MKN-45 cells stably expressing the neomycin resistance gene (MKN-45/control) or Wnt5a (MKN-45/Wnt5a) were subjected to the invasion assay in the presence of Fab fragments or pAb5a-5. Relative invasion activity was expressed as the percentages of the invasion observed by MKN-45/control cells treated with PBS.

(C) The amount of purified Wnt5a bound to mAb5A16 or pAb5a-5 was detected by ELISA. Relative intensities were expressed as arbitrary units.

(D) Top panel, the indicated concentrations of Wnt5a were incubated with control IgG or Fz2CRD-IgG coated on a 96-well plate; the amount of bound Wnt5a was detected by ELISA. Bottom panel, Wnt5a (500 ng/ml) was incubated with control IgG or Fz2CRD-IgG in the presence or absence of 250 μg/ml control Ab, mAb5A16, or 10 μg/ml sFRP2, and then the amount of bound Wnt5a was detected by ELISA. Relative intensities were expressed as arbitrary units.

Results are shown as the mean ± SE of three independent experiments. *, $P < 0.05$.

Figure S2. Wnt5a and Wnt receptor expression levels in HeLaS3, A549, and Calu-6 cells used in this study.

(A) mRNA levels of the indicated genes in cancer cells were examined by semi-quantitative RT-PCR analyses. Results are shown as the fold change compared with mRNA levels in MKN-45 cells (left panel) and shown as the fold increase compared with mRNA levels in KKLS cells (right four panels).

(B-D) Lysates of HeLaS3 cells (B), A549 cells (C), and Calu-6 cells (D) transfected with the indicated siRNAs were probed with the indicated antibodies. HSP90 is used as a loading control.

(E) Lysates of HeLaS3 cells stably expressing the neomycin resistance gene (Control#2) or Wnt5a (Wnt5a#8 or Wnt5a#9) were probed with the indicated antibodies.

(F) Lysates of Control#2 or Wnt5a#8 cells transfected with indicated siRNAs were probed with the indicated antibodies.

(G) Lysates of A549 cells stably expressing GFP or Wnt5a were probed with the indicated antibodies.

(H and I) mRNA levels of the indicated genes in HeLaS3 cells (H) and A549 cells (I) transfected with the indicated siRNAs were examined by semi-quantitative RT-PCR analyses.

Results are shown as the mean ± SE of three independent experiments. *, $P < 0.05$.

Figure S3. Wnt5a signaling is involved in migration and invasion of HeLaS3 and A549 cells.

HeLaS3 and A549 cells transfected with the indicated siRNAs were subjected to the migration (A and C) and invasion (B) assays. Relative migration and invasion activities were expressed as percentages of those in control cells. Results are shown as the mean ± SE of three independent experiments. *, $P < 0.05$.

Figure S4. Wnt5a is required for cell proliferation in KYSE-70 and TE-11 cells.

(A) *Wnt5a* mRNA levels in various cancer cells were examined by semi-quantitative RT-PCR analysis. Results are shown as the fold increase compared with mRNA levels in HeLaS3 cells.

(B) Lysates from KYSE-70 cells and TE-11 cells transfected with Wnt5a#1 siRNA were probed with the indicated antibodies.

(C) KYSE-70 cells (left panel) and TE-11 cells (right panel) transfected with the Wnt5a#1 siRNA were subjected to the proliferation assay.

Results are shown as the mean ± SE of three independent experiments. *, $P < 0.05$.

Figure S5. Labelling of Wnt5a with AlexaFluor 546.

(A and B) Purified Wnt5a (40 pmol) was incubated with (+) or without (-) 200 pmol (A) or 800 pmol (B) of AlexaFluor 546. Labelled (+) or -unlabelled (-) Wnt5a was stained with Coomassie Brilliant Blue (CBB, left top panel) or detected by a fluorescence image analyzer (right top panel). NIH3T3 cells were stimulated with the indicated concentration of labelled (Wnt5a*) or unlabelled (Wnt5a) Wnt5a, and lysates were then probed with anti-Dvl2 antibody (bottom panel).

(C) Colocalization of Wnt5a* with the internalized FLAG-Fz2 was expressed as the percentages of total puncta of the internalized FLAG-Fz2 in HeLaS3 cells.

Results are shown as the mean ± SE of three independent experiments.

Figure S6. Blockade of clathrin-dependent receptor endocytosis suppresses Fz2 internalization in HeLaS3 cells stably expressing Wnt5a.

(A) After transient expression of FLAG-Fz2, HeLaS3 cells stably expressing the neomycin resistance gene (Control#2) or Wnt5a (Wnt5a#8) were pre-treated with 25 μg/ml anti-GST antibody (control Ab) or mAb5A16 for 60 min at 4 °C; then the cells were transferred to a heated chamber (37 °C) for 60 min. The representative confocal images (left panels) and quantification of internalized FLAG-Fz2 (right panels) are shown.

(B) Control#2 or Wnt5a#8 expressing FLAG-Fz2 were pre-treated with 7.5 μM MDC for 48 h for 60 min at 4 °C; then the cells were transferred to a heated chamber (37 °C) for 60 min.

Results are shown as the mean ± SE of three independent experiments. Scale bars, 10 μm. *, $P < 0.05$.

Figure S7. Wnt5a is not required for AKT, PKC, and JNK activities but SFK activities.

(A) Lysates of HeLaS3 cells stably expressing Wnt5a or transfected with Wnt5a siRNAs were probed with the indicated antibodies.

(B) Lysates of HeLaS3, A549, and Calu-6 cells were probed with the indicated antibodies.

(C) HeLaS3 cells were transfected with the indicated siRNAs, and lysates were then probed with the indicated antibodies.

(D) A549 (left top panels) and Calu-6 (right top panels) cells were transfected with the indicated siRNAs, and lysates were then probed with the indicated antibodies. Band intensities of p-SFK at the position of Tyr416 were normalized with band intensities of total Src in each lane (bottom panels). Results are shown as the fold increase compared with the intensity in control cells.

(E) A549 cells stably expressing GFP or Wnt5a (left panels) and Calu-6 cells (right panels) were treated with or without MDC for 48 h, and lysates were then probed with the indicated antibodies.

Results are shown as the mean ± SE of three independent experiments.

Figure S8. Full scan images of immunoblots presented in Figure 2.

(A) Full scan images of immunoblots in Figure 2c.

(B) Full scan images of immunoblots in Figure 2d. Long exposure (top panel) and short exposure (bottom panel) are shown.

Figure S9. Full scan images of immunoblots presented in Figure 4b. Long exposure (top panel) and short exposure (bottom panel) are shown.

Figure S10. Full scan images of immunoblots presented in Figure 5c. Short exposure (top panel) and long exposure (bottom panel) are shown.

Figure S11. Full scan images of immunoblots presented in Figure 6.

(A) Full scan images of immunoblots in Figure 6a.

(B) Full scan images of immunoblots in Figure 6b

Figure S12. Full scan images of immunoblots presented in Figure 6.

(A) Full scan images of immunoblots in Figure 6d.

(B) Full scan images of immunoblots in Figure 6e.

Shojima et al., Figure S1

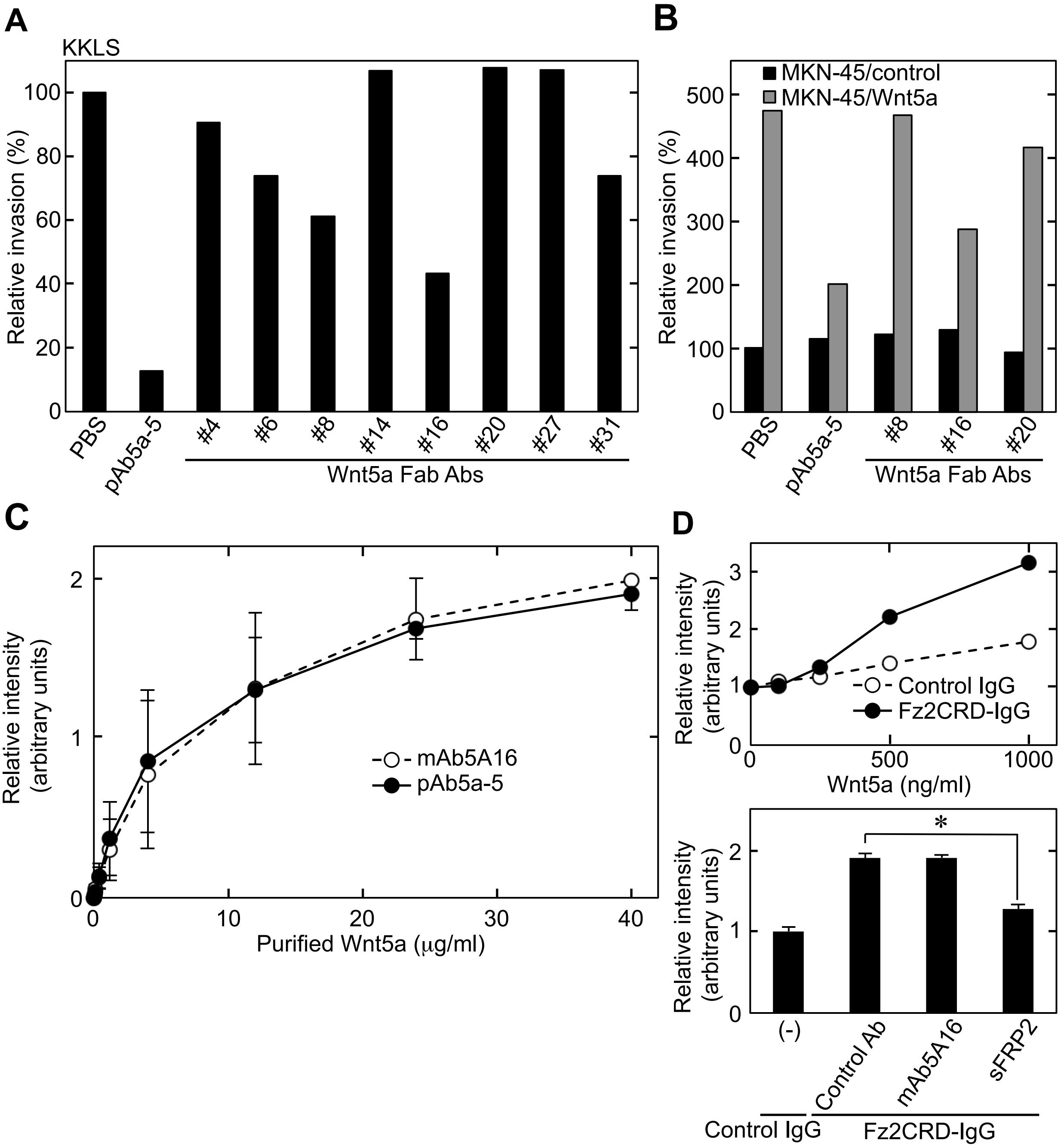

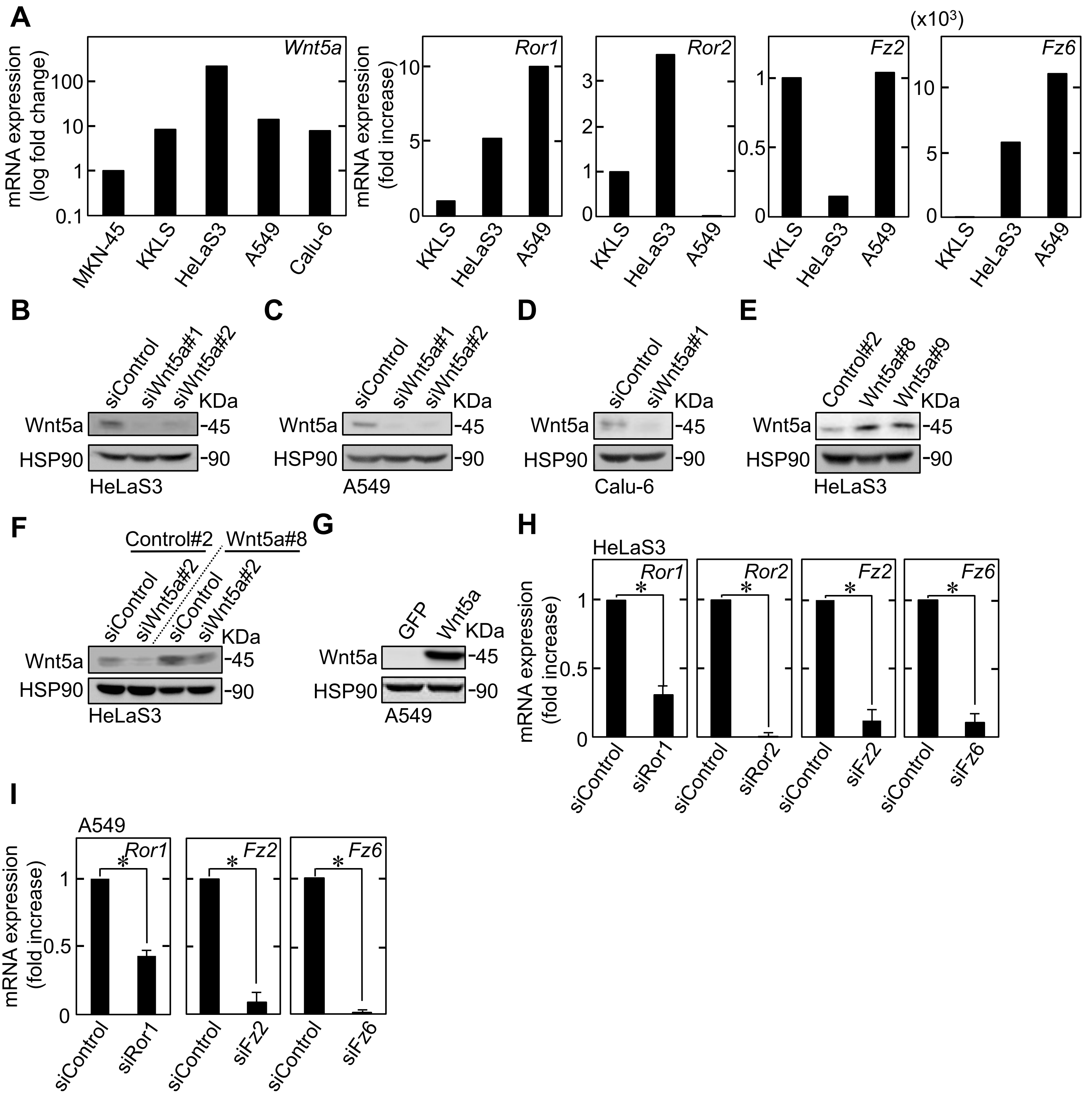
A
mRNA expression (log fold change)
Wnt5a
0.1
1
10
100
MKN-45
KKLS
HeLaS3
A549
Calu-6
mRNA expression (fold increase)
Ror1
Ror2
Fz2
Fz6
(x10³)
KKLS
HeLaS3
A549
B
siControl
siWnt5a#1
siWnt5a#2
KDa
Wnt5a
HSP90
-45
-90
HeLaS3
C
A549
D
Calu-6
E
Control#2
Wnt5a#8
Wnt5a#9
F
Control#2
Wnt5a#8
G
GFP
Wnt5a
H
HeLaS3
siControl
siRor1
siRor2
siFz2
siFz6
I
A549

Shojima et al., Figure S3

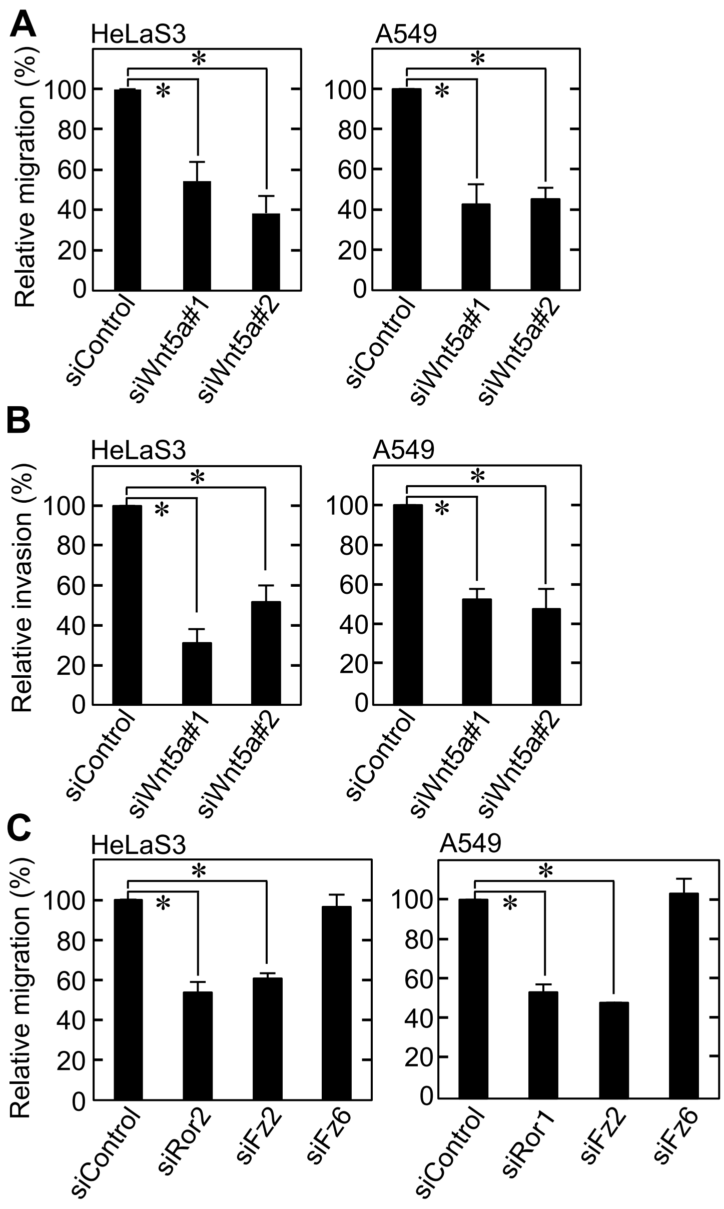

Shojima et al., Figure S4

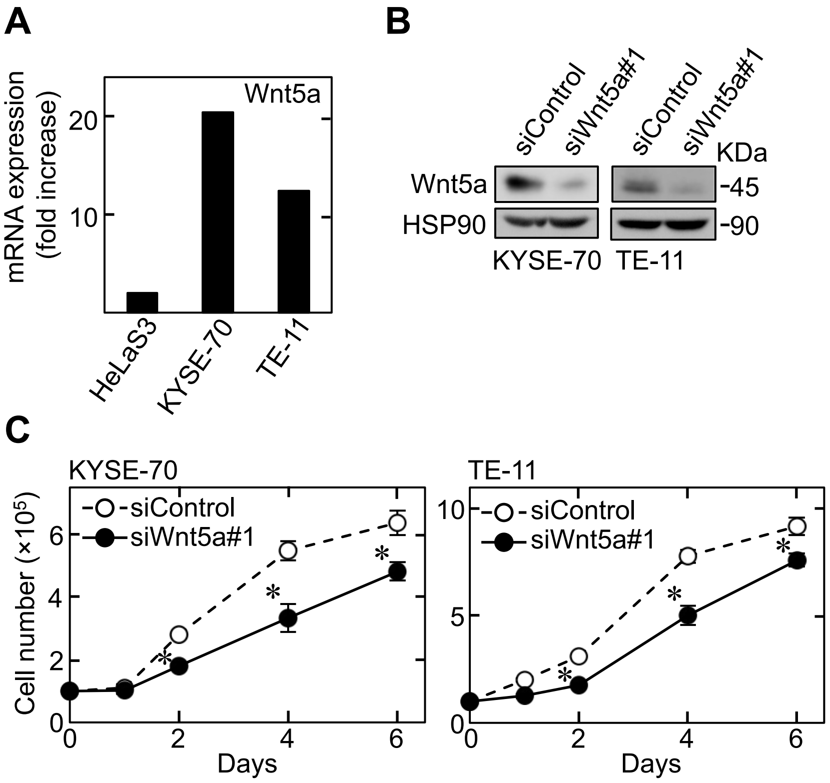

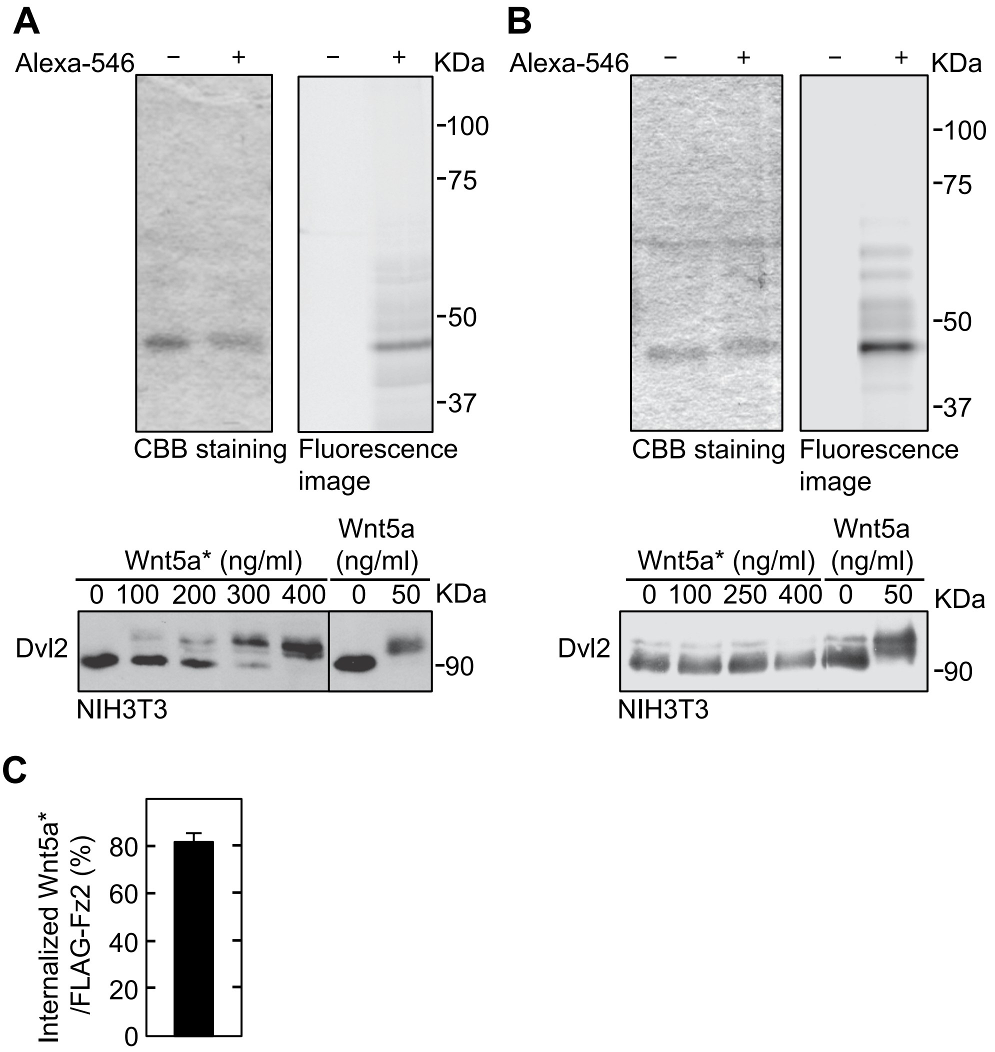
A
Alexa-546
−
+
−
+
KDa
-100
-75
-50
-37
CBB staining
Fluorescence image
Wnt5a* (ng/ml)
Wnt5a (ng/ml)
0 100 200 300 400 0 50
KDa
Dvl2
-90
NIH3T3
B
Alexa-546
−
+
−
+
KDa
-100
-75
-50
-37
CBB staining
Fluorescence image
Wnt5a* (ng/ml)
Wnt5a (ng/ml)
0 100 250 400 0 50
KDa
Dvl2
-90
NIH3T3
C
Internalized Wnt5a* /FLAG-Fz2 (%)
0
20
40
60
80

A

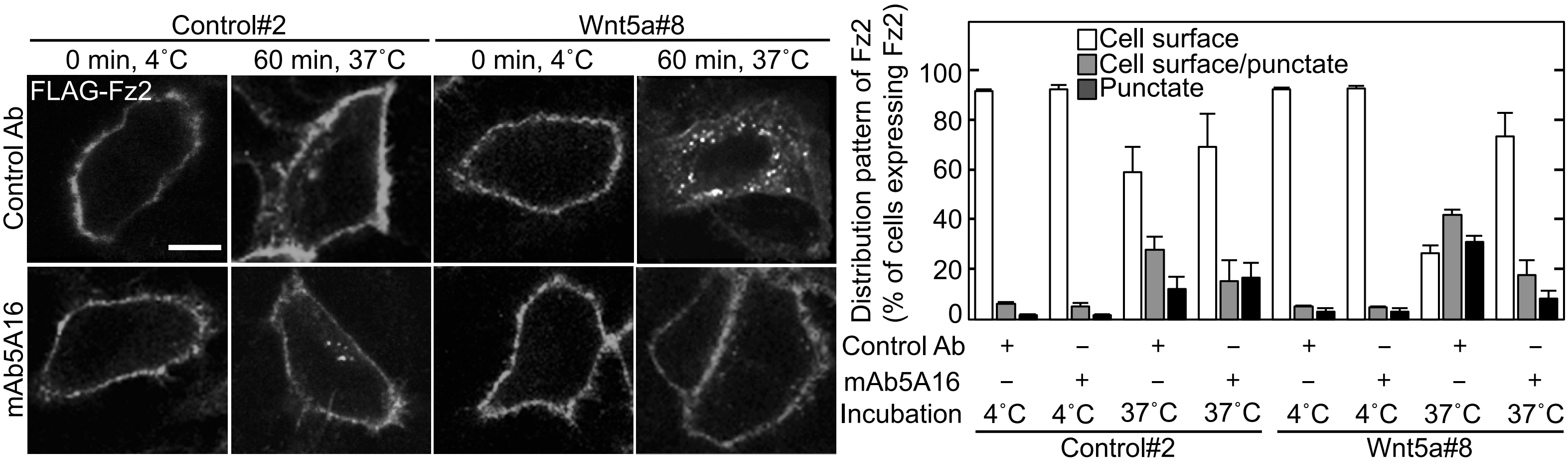
Control#2
Wnt5a#8
0 min, 4˚C
60 min, 37˚C
0 min, 4˚C
60 min, 37˚C
FLAG-Fz2
Control Ab
mAb5A16
Distribution pattern of Fz2 (% of cells expressing Fz2)
Cell surface
Cell surface/punctate
Punctate
100
80
60
40
20
0
Control Ab + – + – + – + –
mAb5A16 – + – + – + – +
Incubation 4˚C 4˚C 37˚C 37˚C 4˚C 4˚C 37˚C 37˚C
Control#2
Wnt5a#8

B

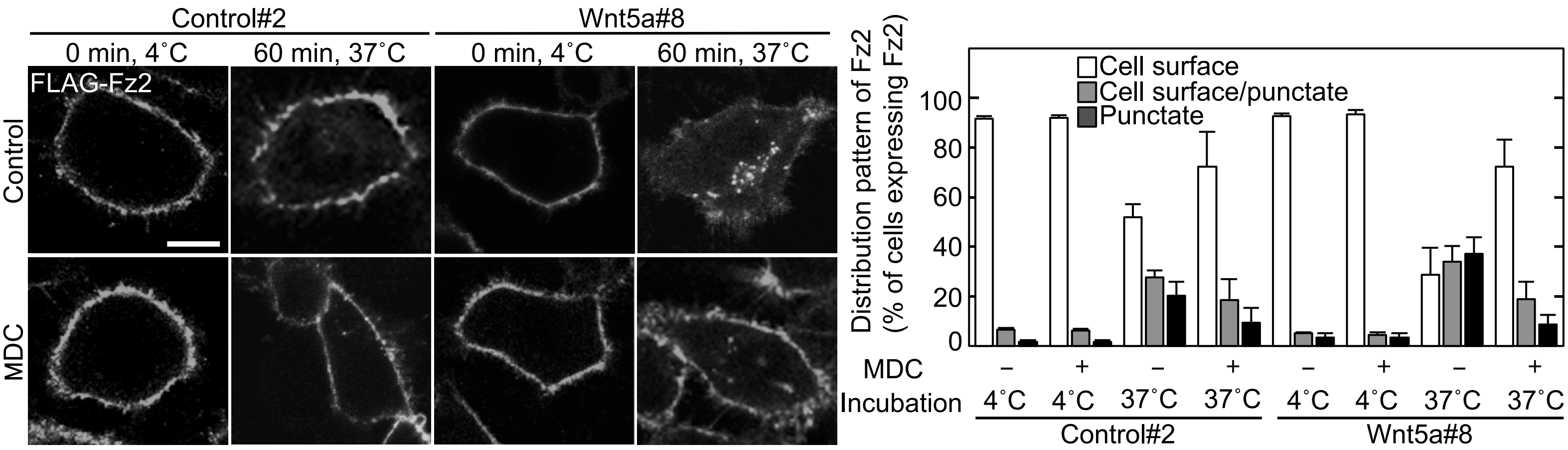
Control#2
Wnt5a#8
0 min, 4˚C
60 min, 37˚C
0 min, 4˚C
60 min, 37˚C
FLAG-Fz2
Control
MDC
Distribution pattern of Fz2 (% of cells expressing Fz2)
Cell surface
Cell surface/punctate
Punctate
100
80
60
40
20
0
MDC – + – + – + – +
Incubation 4˚C 4˚C 37˚C 37˚C 4˚C 4˚C 37˚C 37˚C
Control#2
Wnt5a#8

Shojima et al., Figure S7

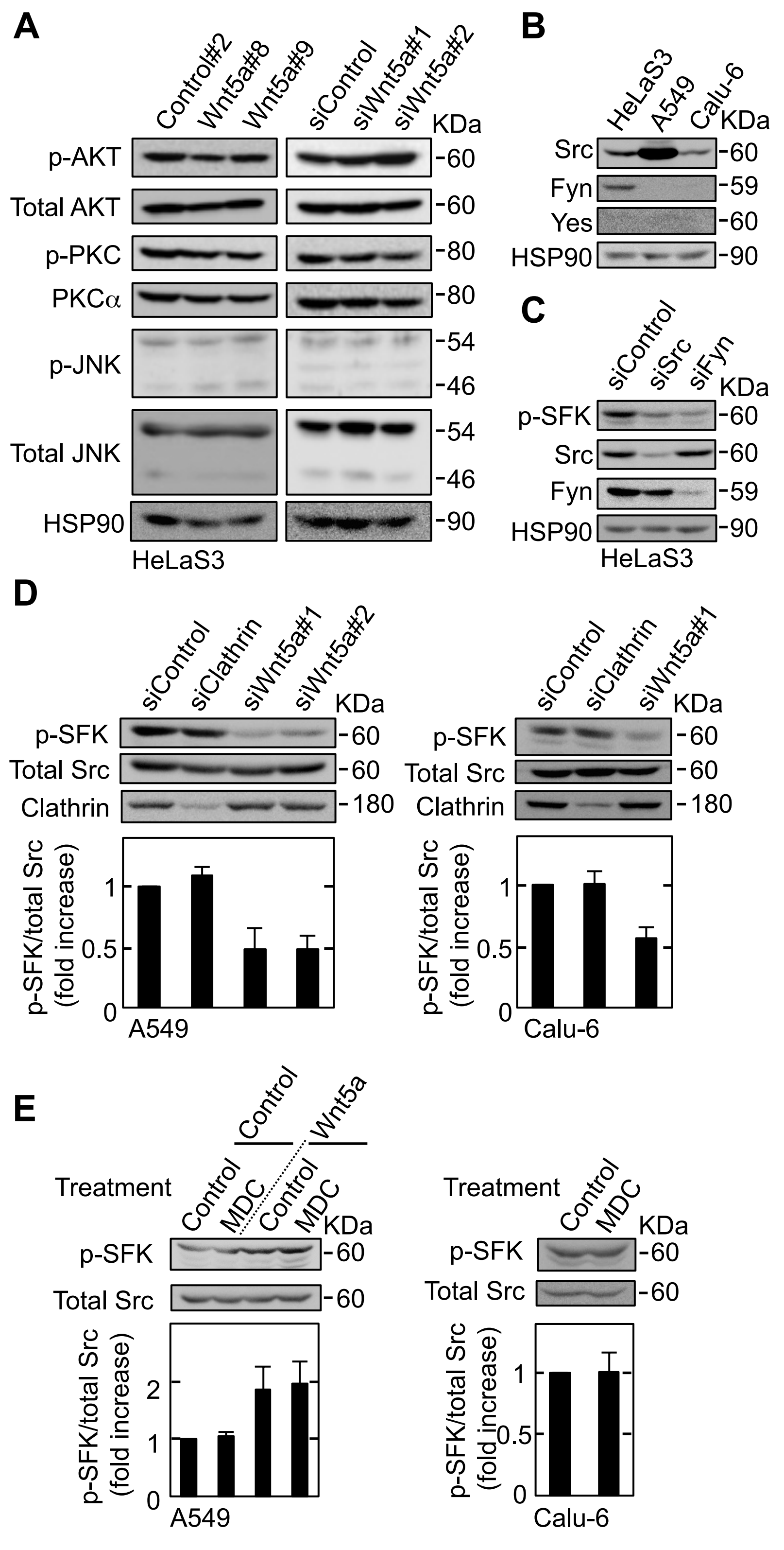

A (Figure 2c)

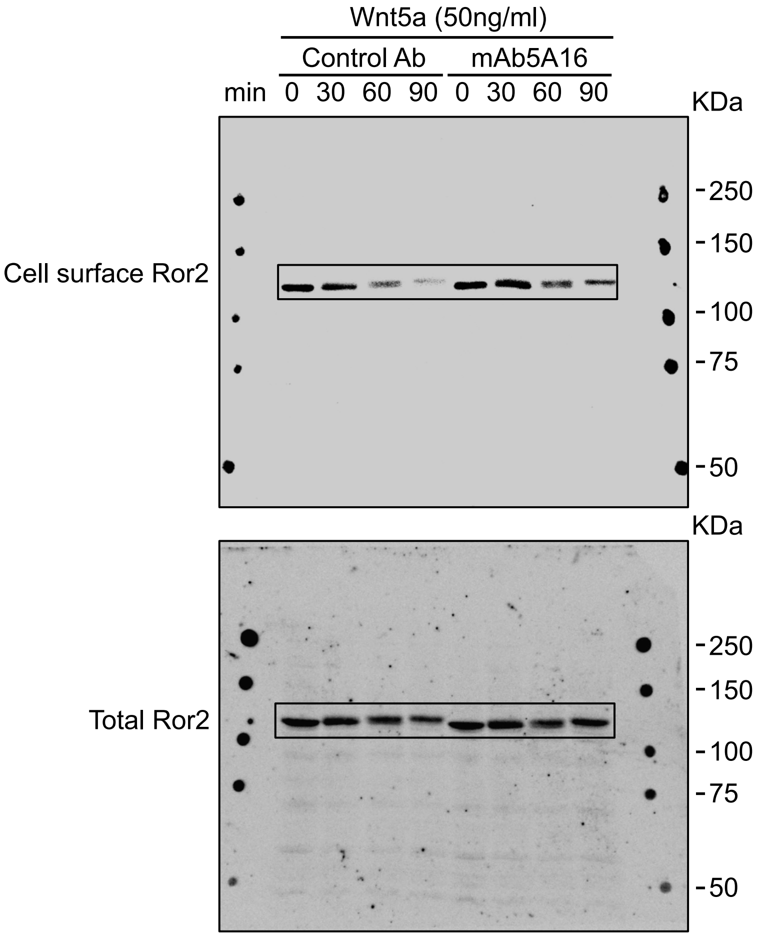

B (Figure 2d)

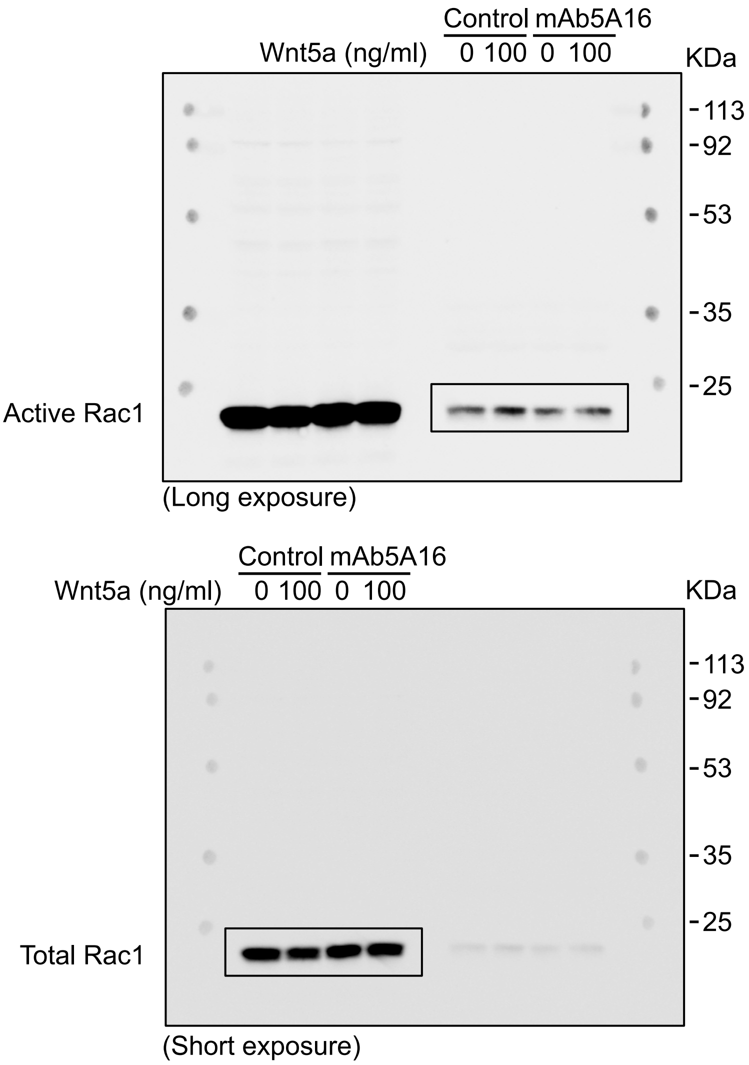

Figure 4b

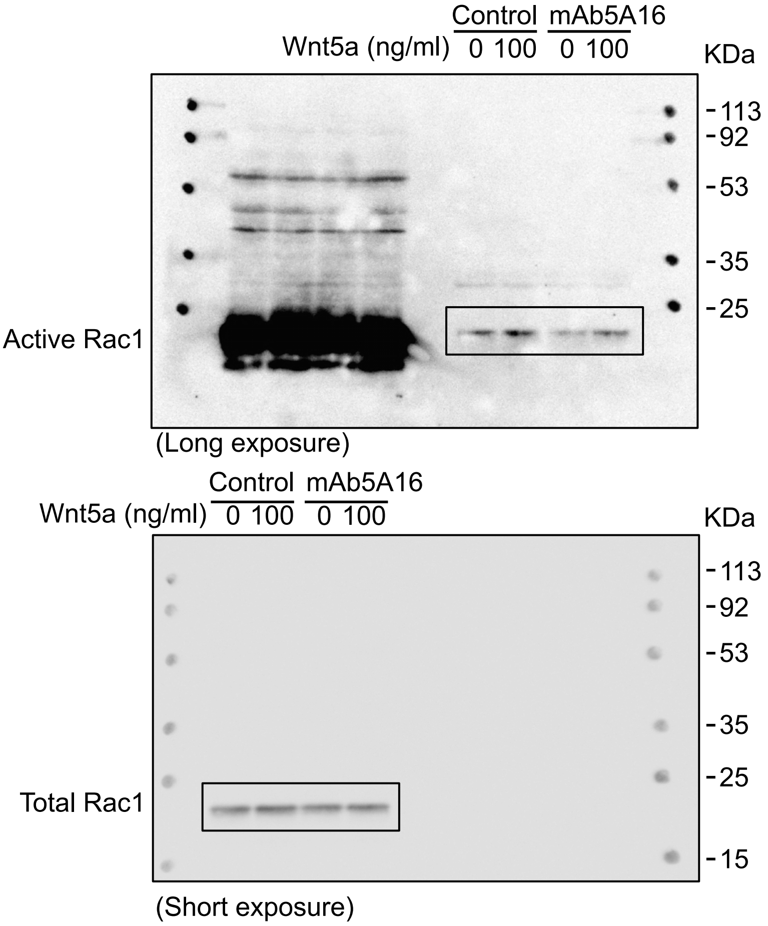
Control
mAb5A16
Wnt5a (ng/ml)
0 100
0 100
KDa
113
92
53
35
25
Active Rac1
(Long exposure)
Control
mAb5A16
Wnt5a (ng/ml)
0 100
0 100
KDa
113
92
53
35
25
15
Total Rac1
(Short exposure)

Figure 5c

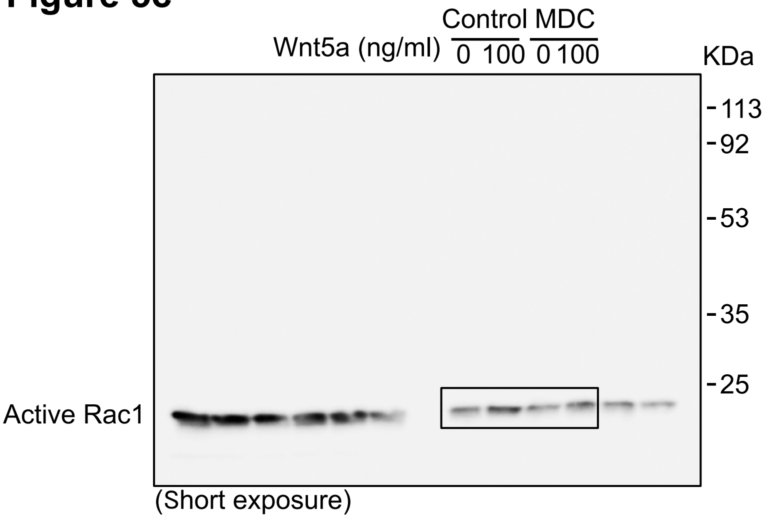
Control MDC
Wnt5a (ng/ml) 0 100 0 100
KDa
113
92
53
35
25
Active Rac1
(Short exposure)

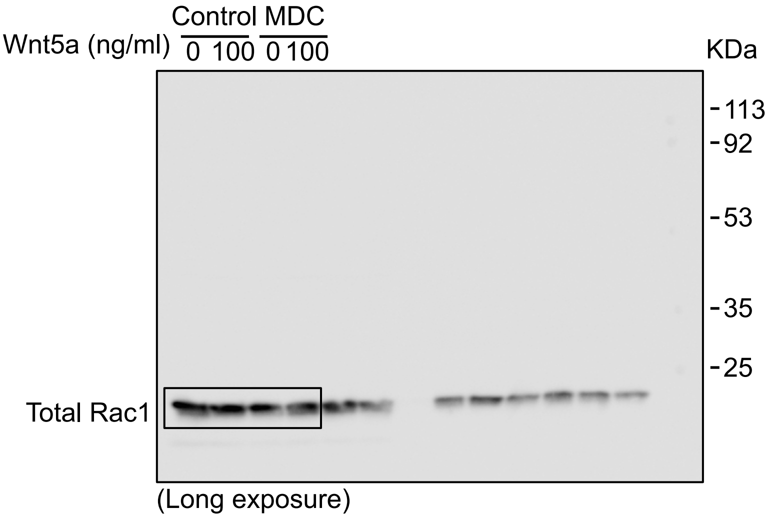
Control MDC
Wnt5a (ng/ml) 0 100 0 100
KDa
113
92
53
35
25
Total Rac1
(Long exposure)

Shojima et al., Figure S11

A (Figure 6a)

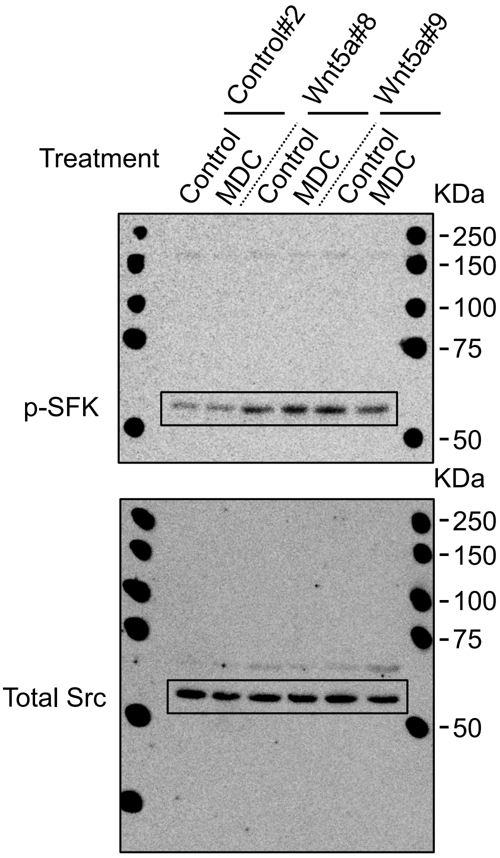

B (Figure 6b)

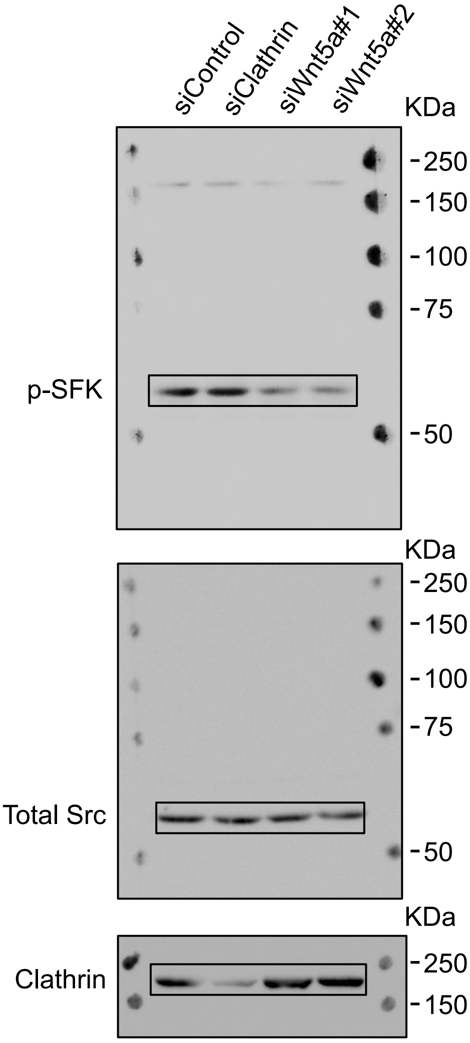

Shojima et al., Figure S12

A (Figure 6d)

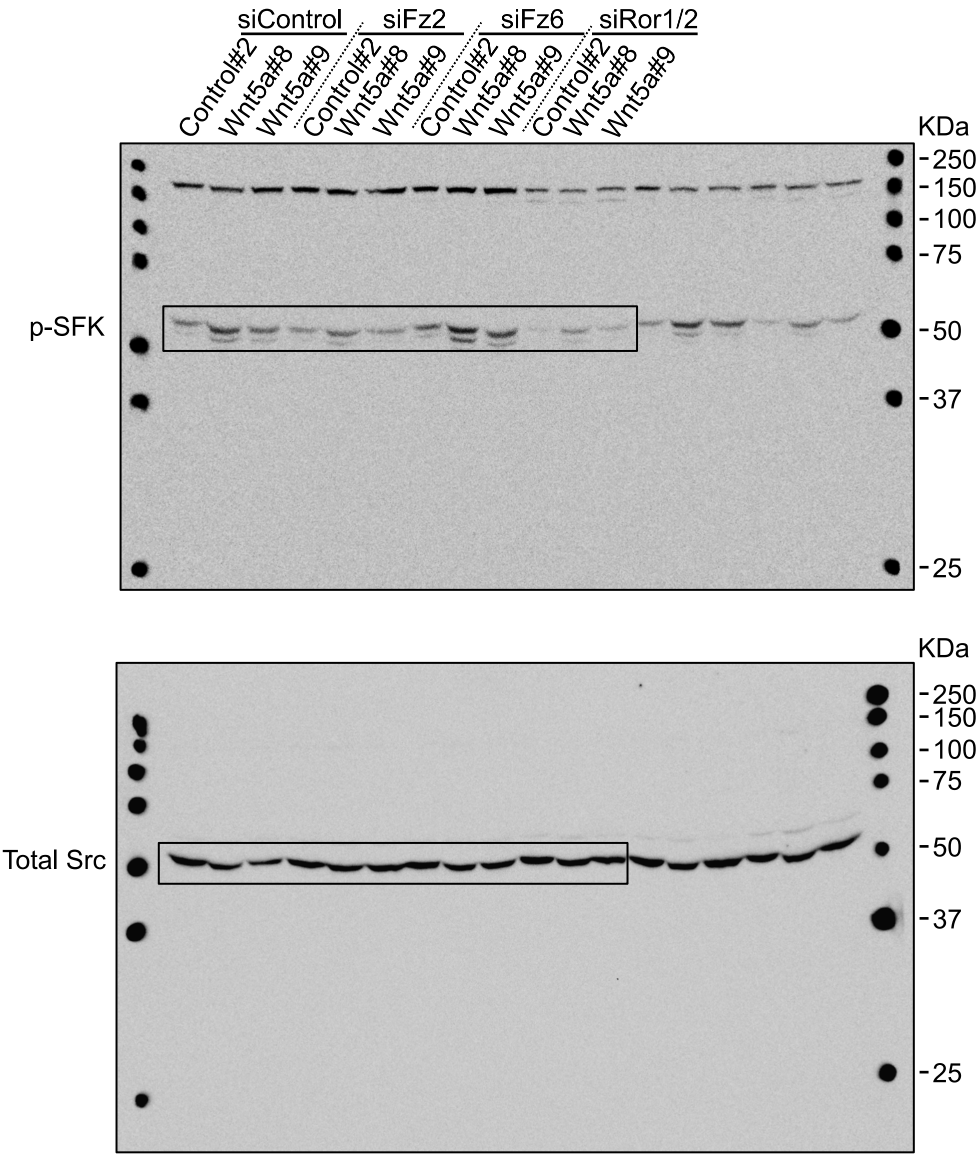

B (Figure 6e)

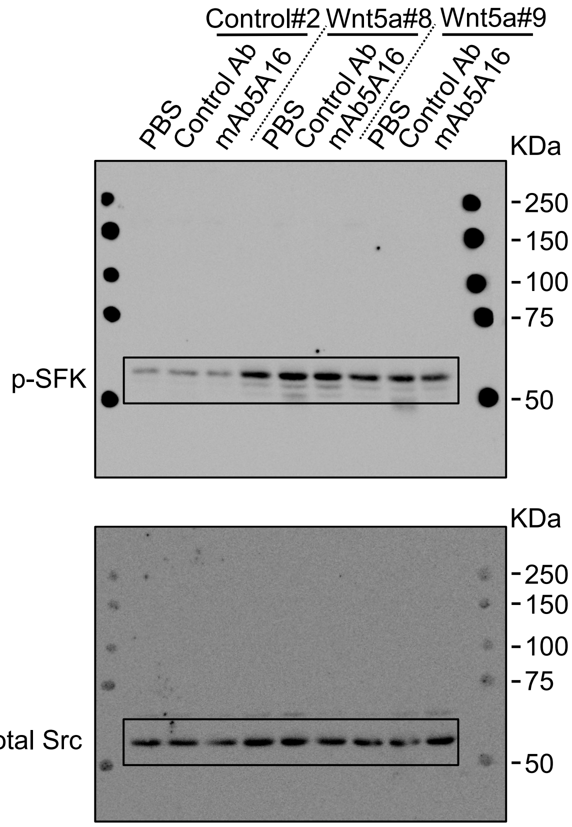

Table S1. Small interfering RNA (siRNA) used in this study

**Target genes**	**Target Sequences**
Randomized control	CAGTCGCGTTTGCGACTGG
Wnt5a	CTGTGGATAACACCTCTGT (#1)<br>CCAAGCTATTTGGAAGCTT (#2)
Ror1	GTACTGCGATGAAACTTCA
Ror2	GGATTACAGAGGAACGGCA
Fz2	CGGTCTACATGATCAAATA
Fz6	GGTTCCACCTTGTCGTAAA
Src	CCTTCCTGGAGGACTACTT
Fyn	GGGATGATATGAAAGGAGA

Table S2. Forward and reverse primers for real-time RT-PCR used in this study

Primer	Sequence
Wnt5a	CTTCGCCCAGGTTGTAATTGAAGC<br>CTGCCAAAAACAGAGGTGTTATCC
Ror1	CAAGGAGGTGGTTTCTTCCA<br>ATTTCACATTCATCGCGACA
Ror2	TGTGTGACGTACCCTCGTGT<br>TGTCCTTCAGCGTTTTGATG
Fz2	GAGCGTGATTGTGCTG<br>GCTCTGGGTAGCGGAA
Fz6	TTCCCTAATCTGATGGGTC<br>TTCAAGCTCCTCAGGC
GAPDH	CCTGTTCGACAGTCAGCCG<br>CGACCAAATCCGTTGACTCC
